# Species‐Specific Responses to Paleoclimatic Changes and Landscape Barriers Drive Contrasting Phylogeography of Co‐Distributed Lemur Species in Northeastern Madagascar

**DOI:** 10.1111/mec.70195

**Published:** 2025-12-10

**Authors:** Tobias van Elst, Dominik Schüßler, Stephan M. Rafamantanantsoa, Tahiriniaina Radriarimanga, Naina R. Rabemananjara, David W. Rasolofoson, R. Doménico Randimbiharinirina, Paul A. Hohenlohe, Ute Radespiel

**Affiliations:** ^1^ Institute of Zoology University of Veterinary Medicine Hannover Hannover Germany; ^2^ Animal Genomics ETH Zurich Zurich Switzerland; ^3^ Institute of Biology and Chemistry University of Hildesheim Hildesheim Germany; ^4^ Mention Anthropobiologie et Développement Durable, Faculté Des Sciences Université D'antananarivo Antananarivo Madagascar; ^5^ Groupe D'étude et de Recherche Sur les Primates de Madagascar (GERP) Antananarivo Madagascar; ^6^ Department of Biological Sciences University of Idaho Moscow Idaho USA

**Keywords:** *A* *vahi*, diversification, gene flow, *Microcebus*, RAD sequencing, river

## Abstract

River barriers have long played a central role in diversification models of tropical regions, including the exceptionally biodiverse island Madagascar. Although their role is best understood by integrating additional factors such as elevation and the ecological niche of a species, empirical studies integrating these variables remain rare. We used restriction site‐associated DNA sequencing to assess the combined effect of rivers, topography, climate and forest cover on the distributions and diversity of four *Microcebus* and two *Avahi* species (Primates, Lemuriformes) in northeastern Madagascar. We inferred population structure, gene flow and genetic diversity, and assessed the association of these ecogeographic variables and genetic differentiation using isolation‐by‐resistance models. Our results show that significant differences in genetic diversity and connectivity among species can be explained by species‐specific responses to landscape features and phylogeographic histories. Specifically, rivers present general barriers to gene flow, but dispersal between inter‐river systems is possible via high‐elevation headwater regions. While this led to high connectivity and genetic diversity in *M. lehilahytsara* and *A. laniger* , gene flow among *M. jonahi* populations is limited by low climatic niche suitability at higher elevations. Moreover, the more restricted distributions of *M. macarthurii* , *M. simmonsi* and *A. mooreorum* likely resulted from refugial dynamics and sea level fluctuations leading to allopatric divergence and microendemism. Together, the findings illustrate how ecological differences among species and temporal landscape dynamics mediate the role of rivers as dispersal barriers. They also highlight the importance of prioritising river headwaters and topographically complex regions, which were shown to promote connectivity, in conservation efforts.

## 1 Introduction

The mechanisms and factors underlying the diversification of species are of central importance to evolutionary biology. Speciation typically results from a restriction of gene flow between populations, causing differentiation due to genetic drift and selection (Coyne and Orr 2004; Mayr 1942). The spatial separation of populations by geographic barriers like water bodies, mountain ranges or otherwise disconnected habitat is considered the predominant mode of speciation in animals (allopatric speciation; Coyne and Orr 2004). However, a restriction of gene flow can also occur more gradually. For instance, isolation‐by‐distance explains population structure in many species (Sexton et al. 2014). Dispersal can similarly be limited through (continuous) landscape features other than distance (isolation‐by‐resistance; IBR; McRae et al. 2008). Divergence along such ecogeographic gradients (parapatric speciation) is also well documented (e.g., Fenker et al. 2021; Jaynes et al. 2022; Myers et al. 2019).

Rivers have played a central role in allopatric speciation models ever since Wallace (1852) first hypothesised the importance of the Amazon River in explaining species distributions and divergences. Since then, the riverine barrier hypothesis has been tested in a wide range of taxa in Amazonia (e.g., Boubli et al. 2015; Patton et al. 2000; Peres et al. 1996) and other regions worldwide (e.g., continental Africa: Anthony et al. 2007; Madagascar: Olivieri et al. 2007; Pastorini et al. 2003; Southeast Asia: Klabacka et al. 2020). It is now evident that rivers and their size alone present a relatively poor explanation for many biogeographic patterns and divergence processes. Rather, their effect needs to be modeled alongside additional ecogeographic features, such as elevation and the ecological niche of a species, which modulates the effect of a landscape on dispersal and gene flow (e.g., Janiak et al. 2022; Kopuchian et al. 2020; Naka and Pil 2020). For instance, headwater regions may provide dispersal corridors across river barriers depending on the dispersal ability and elevational tolerance of a species (Goodman and Ganzhorn 2004; Haffer 1997). In addition, rivers are best modeled as dynamic geographic features as their course and discharge vary over time, changing the availability of suitable habitat and paleoclimatic refugia, which can promote divergences as well (Clementucci et al. 2025; Schüßler, Bremer, et al. 2025; Wilmé et al. 2006).

Madagascar is uniquely suited to study the ecogeographic factors that modulate riverine barrier effects as it is one of the most biodiverse places on Earth with a complex physical geography characterised by extensive river systems across elevational gradients (Antonelli et al. 2022; Goodman 2022; Vences et al. 2009). The evolution of the island's endemic biota has been predominantly attributed to allopatric and parapatric speciation driven by geographic barriers and cycles of isolation to glacial refugia and subsequent range expansion, particularly during the Quaternary (e.g., watershed model; Mercier and Wilmé 2013; Wilmé et al. 2006; reviewed in Brown et al. 2014; Vences et al. 2009). Paleoclimatic dynamics could also have led to the fragmentation and divergence of populations due to sea level changes (sea levels at glacial maxima were up to 120 m lower than today; Bintanja et al. 2005). While previous work in Madagascar has compared observed diversity patterns with predictions from barrier and refugia models both on the species‐ and community level (e.g., Brown et al. 2014, 2016; Everson et al. 2020; Pastorini et al. 2003; Scherz et al. 2023; Schüßler, Bremer, et al. 2025), explicit models integrating the effect of rivers and other landscape features remain rare (but see Baden et al. 2019).

We selected a region in northeastern Madagascar (hereafter referred to as the study region) located between Marojejy National Park (NP) and Betampona Special Nature Reserve (SNR; Figures S1 and S2; Poelstra et al. 2021) to explicitly model the interplay of ecogeographic diversification factors in six lemur species. Harbouring some of the island's largest remaining continuous humid forests (both high‐ and lowland; Vieilledent et al. 2018), almost one fourth of all lemurs, a radiation of more than 100 species across five extant families, can be found in the study region (Mittermeier et al. 2023). This includes four of the 19 currently recognised mouse lemur species (genus *Microcebus*; *M. jonahi, M. macarthurii, M. lehilahytsara* and *M. simmonsi* ; Poelstra et al. 2021; Schüßler, Blanco, et al. 2020; Tiley et al. 2022; van Elst et al. 2025) and two of the nine described woolly lemur species (genus *Avahi*; *A. laniger* and *A. mooreorum* ; Lei et al. 2008; Zaramody et al. 2006). In addition, Louis and Lei (2016) hypothesised the presence of another yet undescribed *Microcebus* species (*M*. sp. #2) on the Masoala peninsula based on differentiation in mitochondrial genome sequences. The study region is characterised by an elevational cline from the Central Highlands towards the east coast, with rivers of different sizes separating it latitudinally into 17 inter‐river systems (IRSs; Figure S1). The basin of one river, the Antainambalana River, was previously hypothesised to have served as a retreat‐dispersion watershed for species during Pleistocene climatic fluctuations (Mercier and Wilmé 2013; Wilmé et al. 2006). In addition, the island Île Ste. Marie (Nosy Boraha) lies on the continental shelf about 10 km away from the mainland and was likely connected to it during times of low sea levels such as the Last Glacial Maximum (Bintanja et al. 2005; Rohling et al. 2022). Ecological and genetic work (Andriantompohavana et al. 2007; Poelstra et al. 2021; Schüßler, Blanco, et al. 2020; Tiley et al. 2022; Zaramody et al. 2006) indicated that lemur species in the region differ greatly in their distributional areas and patterns of diversity, with *M. lehilahytsara* and *A. laniger* encompassing much larger distributions than other species in their genera. Due to their interspecific focus, however, the studies relied on few sampling sites per species, leaving most IRSs unsampled. Consequently, the intraspecific diversity, population structure and particularly the determinants of population differentiation of species in the region remain open questions.

Here, we assessed the effect of rivers and additional ecogeographic factors on the distributions and diversity of four *Microcebus* and two *Avahi* species in the study region ( *M. jonahi* , *M. macarthurii* , *M. lehilahytsara* , *M. simmonsi* , *A. laniger* , *A. mooreroum*), based on restriction site‐associated DNA (RAD) sequencing of 344 individuals. Specifically, we inferred population structure, gene flow and genetic diversity, and used isolation‐by‐resistance models to assess the species‐specific roles of topography, rivers, climate and forest cover in shaping genetic differentiation. We interpret our findings in light of Madagascar's dynamic paleoclimatic history and ongoing threats to biodiversity.

## 2 Material and Methods

### 2.1 Sampling

We aimed to cover the entire distributions of the six study species in the region in our sampling by including high‐ and lowland sites in each IRS if possible (Figures S1–S3). Between 2006 and 2022, we collected a total of 269 and 34 individual ear biopsies at 33 and 11 different localities following Radespiel et al. (2008) and Hokan et al. (2017) for the genera *Microcebus* and *Avahi*, respectively (Table S1). Our sampling significantly improves our knowledge on the distributions of all six species (see Results in Data S1). Ear biopsies of four *A. occidentalis* outgroup individuals were also included. We complemented our data set with published sequences of 104 *Microcebus* individuals, including *M. lehilahytsara* populations south of the study region and *M. murinus* outgroups (Poelstra et al. 2021; Tiley et al. 2022; Table S1).

### 2.2 RAD Sequencing and Genotyping

We generated single‐digest restriction site associated DNA sequencing (RADseq) libraries using the enzyme *SbfI*. To do so, we extracted genomic DNA with a modified QIAGEN DNeasy Blood & Tissue Kit protocol (see Sgarlata et al. 2018). New RADseq libraries were prepared and paired‐end sequenced to a target coverage of 15× following two different protocols (see Methods in Data S1 and Table S2 for details). Sequencing data were demultiplexed and adapters were trimmed with bcl2fastq v2.20 (Illumina, Inc.), discarding reads with final length smaller than 20 bases. As mentioned, we added published RADseq data of 104 *Microcebus* samples (Table S1; Poelstra et al. 2021; Tiley et al. 2022). For these, adapters were trimmed using Trimmomatic v0.39 (Bolger et al. 2014) with the following parameters: Leading: 3, Trailing: 3, Slidingwindow: 4:15, Minlen: 60.

Trimmed reads of *Microcebus* individuals were aligned against the *M. murinus* reference genome Mmur 3.0 (Larsen et al. 2017) with BWA‐MEM v0.7.17 (Li and Durbin 2009), retaining only those that mapped to autosomal scaffolds, had a mapping quality larger than 20 and were properly paired. Subsequently, we used the reference‐based approach of Stacks v2.53 (Rochette et al. 2019) to call genotypes across individuals. For the genus *Avahi*, the *de novo* approach of Stacks was used to build a catalogue and call genotypes from trimmed reads of individuals with the parameters *M* = 2, *m* = 2 and *n* = 3 due to the lack of a reference genome. Parameter tuning was conducted following Paris et al. (2017). In this way, we created two genus‐specific genotype call sets for phylogenetic inference and to compare estimates of genetic diversity as well as four (sister) species‐specific sets ( *M. jonahi* + *M. macarthurii* , *M. lehilahytsara* , *M. simmonsi* and *A. laniger* + *A. mooreorum* ) to increase signal and enable more accurate filtering for population genomic analyses. All sets were filtered following FS6 filtering recommendations of O'Leary et al. (2018) with modified thresholds and a minor allele count filter of 3 (see Methods in Data S1), using VCFtools v0.1.17 (Danecek et al. 2011).

### 2.3 Phylogenetic Inference

We performed maximum likelihood phylogenetic inference on the genus‐specific genotype call sets, using the GTR + Γ model of sequence evolution in IQ‐TREE v2.2.0 (Minh et al. 2020) while correcting for ascertainment bias. We estimated ultrafast bootstrap support and performed a Shimodaira‐Hasegawa‐like approximate likelihood ratio test (SH‐aLRT; Guindon et al. 2010), using 1000 replicates.

### 2.4 Population Genetic Structure and Gene Flow

We investigated population structure through clustering and principal component analysis (PCA). Clustering probabilities were inferred via maximum likelihood in ADMIXTURE v1.3.0 (Alexander et al. 2009), setting *K* from one to a maximum of 14. Optimal *K* was estimated via cross validation. PCA was conducted in the R package ‘SNPRelate’ v1.32.2 (Zheng et al. 2012).

We inferred directional rates of gene flow between adjacent populations under a coalescent model with the R package ‘gene.flow.inference’ v0.0.0.9 (Lundgren and Ralph 2019). As input to the model, we used the genetic distance *D*_*PS*_ (= 1—proportion of shared alleles; Bowcock et al. 1994) between populations. We ran the algorithm for a total of 8,000,000 generations with 12.5% pre‐burn‐in and 37.5% burn‐in.

We also tested for excess allele sharing between *M. macarthurii* (IRS 5) and adjacent *M. jonahi* (IRS 6) as well as between *A. mooreorum* (IRSs 4 and 2/3) and adjacent *A. laniger* (IRS 5) with Patterson's *D*‐statistic (Patterson et al. 2012), as implemented in Dsuite v0.4 r38 (Malinsky et al. 2021). We used *M. lehilahytsara* and *A. occidentalis* as outgroups, respectively, and based the test on 15 randomly selected individuals per clade (or all individuals if fewer than 15 were available).

### 2.5 Drivers of Population Genetic Structure

We first tested for the presence of isolation‐by‐distance in the four species *M. jonahi* , *M. lehilahytsara* , *M. simmonsi* and *A. laniger* , which can be considered the null model in landscape genetic analyses (Balkenhol et al. 2009; Jenkins et al. 2010). To do so, we performed a Mantel test based on Spearman's rank correlation *r*_s_ of genetic vs. geographic distances among individuals as well as populations with 9999 permutations in the R package ‘vegan’ v2.5‐7 (Dixon 2020). As a measure of genetic distance, we again used *D*_PS_ because it is less likely to be biased by unequal sample size and/or violations of assumptions than the traditionally used *F*_ST_ (Peterman et al. 2014). Spatial deviations from a model of isolation‐by‐distance were visualised with Estimated Effective Migration Surfaces (EEMS; Petkova et al. 2015), which was run for 4,000,000 generations with a burn‐in of 25% and using 1000 demes. Unlike in the inference of population structure, diversity and gene flow, *M. macarthurii* and *A. mooreorum* were not included alongside their sister species in these analyses given that intrinsic reproductive barriers could bias inference.

We then evaluated the relationship between five ecogeographic landscape variables potentially affecting lemur dispersal (elevation, landscape heterogeneity, rivers, climatic niche models and forest cover) and genetic distances *D*_PS_ between individuals through isolation‐by‐resistance models for the four species *M. jonahi* , *M. lehilahytsara* , *M. simmonsi* and *A. laniger* (see Methods in Data S1 and Table S1 for sample selection). Models were optimised with the gradient‐based algorithm of the R package ‘radish’ v0.0.2 (Peterman and Pope 2021), and their likelihood was inferred through multiple regression of landscape resistances with genetic distances, while allowing diagonal movement. Specifically, we used a log‐linear conductance model and a maximum likelihood population effect parameterization for linear mixed‐effect models (MLPE), which account for the non‐independence of pairwise landscape and genetic distances, therefore providing an accurate approach for model inference based on individual distances (Clarke et al. 2002; Shirk et al. 2018; van Strien et al. 2014). We compared a total of 26 different models per species based on the Akaike Information Criterion (AIC), including the null model of IBD, all univariate models and multivariate models with up to three variables (Tables S4–S7). Models with four or more predictors were not compared as many of these failed to reach convergence. Spatial rasters for the five predictor variables were created at a spatial resolution of 150 m as follows:

1. Elevation (Figure S4) was encoded as a continuous variable based on the digital elevation model by GEBCO Compilation Group (2023).
2. Topographic complexity (Figure S5) was encoded as a continuous variable by calculating landscape heterogeneity from the same digital elevation with the R package ‘rasterdiv’ v0.3.4 (Rocchini et al. 2021). In brief, this metric is based on a sliding window approach that assigns to each pixel an estimate of the variation among adjacent pixels.
3. Rivers (Figure S6) were encoded as a continuous variable using flow accumulation, that is the amount of water that accumulates in a given area, as a proxy for size (O'Callaghan and Mark 1984). Flow accumulation was calculated from the same digital elevation model with the R package ‘whitebox’ v2.2.0 (Lindsay 2016). Rivers were padded to a width of two pixels to avoid diagonal traversal of rivers.
4. Climatic niche models (Figures S7–S10) were inferred from spatially filtered occurrence records for each species (Table S3) and based on eight bioclimatic variables with ecological relevance for lemurs (see Methods in Data S1 for details).
5. The oldest available forest cover estimates of the region (i.e., from 1953) were obtained from Vieilledent et al. (2018) to approximate forest vegetation prior to human impact as accurately as possible (Figure S11). Human pressure and associated habitat degradation in the region started only relatively recently (Ralimanana et al. 2022; Schüßler, Mantilla‐Contreras, et al. 2020; Vieilledent et al. 2018). Unpublished data by J. Salmona et al. indicate that these earliest estimates best explain the population structure of another lemur species, *Propithecus coquereli* , across its range.

All raster layers were cropped to the focal region of each species with a buffer of 35 km, centred around 0 and scaled by the standard deviation, using the R package ‘terra’ v 1.6.47 (Hijmans 2022). Correlation coefficients among raster layers were below 0.7, based on a pixel subsample of 5% (Figure S12).

### 2.6 Genetic Diversity

To infer how genetic diversity is distributed across space and to identify potential glacial refugia, we estimated individual observed heterozygosities *H*_O_ from the genus‐specific genotype call sets with VCFtools. Former refugia are expected to harbour populations with comparably high genetic diversity, which decreases in the direction of postglacial range expansion (e.g., due to founder effects; Hewitt 1996, 1999). We quantified the relationships between *H*_O_ and elevation, latitude and longitude in *M. jonahi* , *M. lehilahytsara* , *M. simmonsi* and *A. laniger* via Spearman's rank correlation in R to identify potential glacial refugia (following Bagley et al. 2017). The correlation was not estimated in *M. macarthurii* and *A. mooreorum* due to the limited sample sizes. We also tested for significant differences between species of the same genus with a Kruskal–Wallis test and Dunn's (post hoc) test with Bonferroni correction.

## 3 Results

### 3.1 RADseq and Genotyping Statistics

We sequenced an average of 9,342,093 (SD = 5,249,265) raw reads per sample, resulting in 80,560 (SD = 11,971) recovered RAD loci across *Microcebus* samples and 130,696 (SD = 27,188) loci across *Avahi* samples, with mean coverages of 10.86× (SD = 6.05×) and 9.98× (SD = 3.98×), respectively (Table S2). Two *Avahi* individuals from IRS 11 were excluded prior to genotyping due to low sequencing quality. While the genus‐specific SNP sets included 157,306 (*Microcebus*) and 27,100 (*Avahi*) filtered variant sites, the (sister) species‐specific SNP sets comprised 45,865 ( *M. jonahi* + *M. macarthurii* ), 112,138 ( *M. lehilahytsara* ), 25,838 ( *M. simmonsi* ) and 28,965 ( *A. laniger* + *A. mooreorum* ) variants after filtering. Proportions of missing data are given in Table S2.

### 3.2 Phylogenetic Inference

**FIGURE 1:**
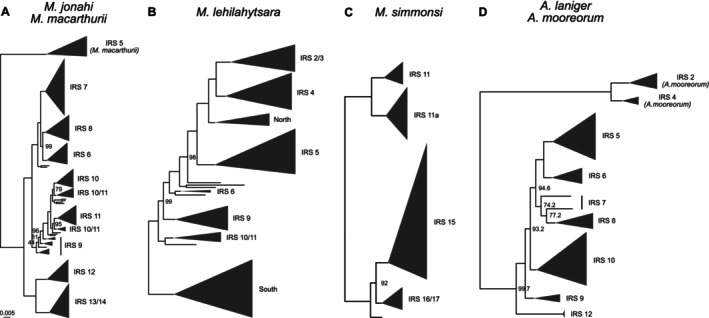
Maximum likelihood phylogenies inferred with IQ‐TREE for *M. jonahi/M. macarthurii* (A), *M. lehilahytsara* (B), *M. simmonsi* (C) and *A. laniger* / *A. mooreorum* (D). Tip labels denote inter‐river systems (IRSs). ‘North’ and ‘South’ correspond to *M. lehilahytsara* populations north (Anjanaharibe‐Sud Special Reserve and Marojejy National Park) and south (see Tiley et al. 2022) of the study region, respectively. Triangles represent collapsed tips proportional to sample size. Node labels represent ultrafast bootstrap support, given only for nodes between major clades and if below 100. Scale is substitutions per site. Detailed phylogenies for each species are given in Figures S13–S16.

*M. macarthurii* (IRS 5) and *M. jonahi* (IRSs 7–14) were recovered as reciprocally monophyletic (Figure 1, Figure S13). The deepest split in the *M. jonahi* phylogeny coincided with the Simianona River. The lineage north of this river formed two well‐supported clades, one comprising populations from IRSs 6–8 and one comprising those from IRSs 9–11 (separated by the Fahambahy River). Similarly, the well‐supported southern lineage exhibited a split between populations from IRSs 12 and 13/14, respectively (separated by the Marimbona River). More recent divergences were difficult to resolve, and support values were lower.

As already shown in Tiley et al. (2022), *M. lehilahytsara* populations from central eastern and northeastern Madagascar formed two distinct clades (Figure 1, Figure S14). Populations of the northern clade divered sequentially from south to north with strong statistical support, startig with IRSs 10 and 11, followed by IRS 9, 6, 5, 4 and 2/3. Individuals from Masoala (IRS 2/3), which have been proposed to represent a distinct species by Louis and Lei (2016), were nested within the *M. lehilahytsara* phylogeny. Highland samples from the far north of the study region (Anjanaharibe‐Sud Special Reserve (SR) and Marojejy NP) clustered as sister to IRSs 2/3 and 4.

For *M. simmonsi* , we recovered one major clade formed by the northern populations in IRS 11 and on Île Ste. Marie (IRS 11a) and one comprising the southern populations (IRSs 15–17; Figure 1, Figure S15).

The divergence between *A. mooreorum* (IRSs 2 and 4) and *A. laniger* (IRSs 5–12) was well supported (Figure 1, Figure S16). Similar to *M. jonahi* , the Simianona River corresponded to the deepest split in the *A. laniger* phylogeny. Among populations north of this river, the population from IRS 9 represented the earliest divergence, followed by those from IRS 10 and a clade composed of sister lineages corresponding to IRSs 7 and 8 and IRSs 5 and 6, respectively.

### 3.3 Population Genetic Structure and Gene Flow

**FIGURE 2:**
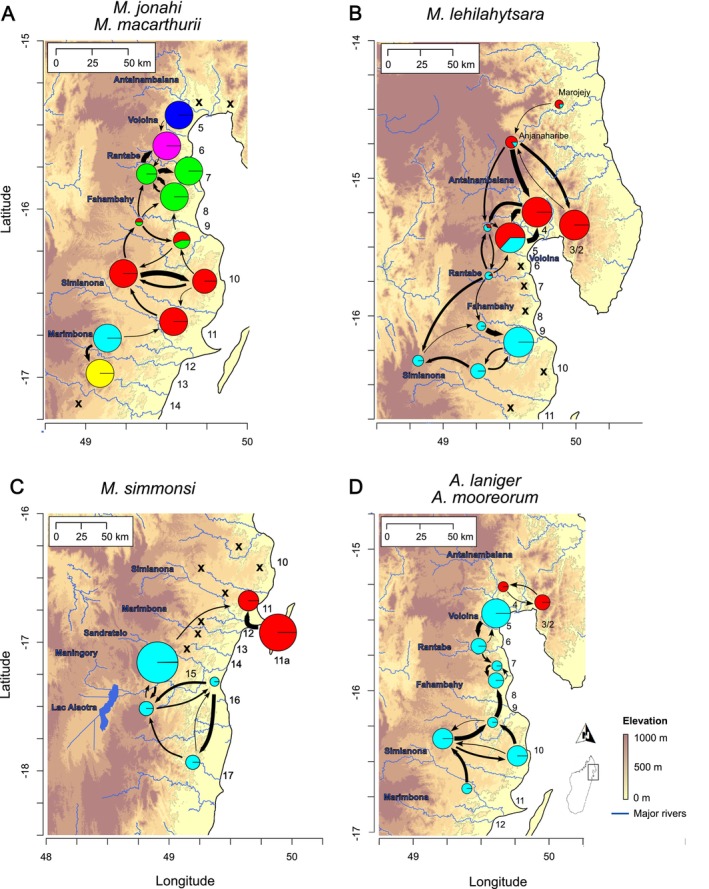
Population structure of *M. jonahi/M. macarthurii* (A), *M. lehilahytsara* (B), *M. simmonsi* (C) and *A. laniger* / *A. mooreorum* (D) in the study region in northeastern Madagascar. Coloured pie charts show clustering proportions of sampled populations based on the best number of clusters (*K* = 6 for *M.jonahi*/ *M. macarthurii* ; *K* = 2 for the remaining taxa). In panel A and D, the dark blue and red pie charts correspond to *M. macarthurii* and *A. mooreorum* , respectively. Pie chart size is proportional to sample size. Arrows indicate major gene flow between adjacent populations. Arrow width is proportional to relative gene flow rates (see Tables S8–S11 for details). Please note that arrows represent gene flow but not necessarily migration routes (e.g., across a river). Crosses indicate absence records. Numbers denote inter‐river systems. Blue labels denote names of rivers and Lac Alaotra.

Cross‐validation of clustering analysis supported *K* = 6 as the best number of clusters for the set of *M. jonahi* and *M. macarthurii* individuals (Figure S17). These clusters largely corresponded to phylogenetic clades and the regions between rivers with the highest‐elevation headwaters (e.g., Rantabe, Simianona and Marimbona Rivers; Figure 2A, Figure S18). Individuals in IRS 9, which is formed by rivers with low‐elevation headwaters, were assigned equally high probabilities to belong to clusters of populations north and south of them. This pattern disappeared at *K* ≥ 12. Clustering results were also supported by PCA (Figure S19), in which clusters were clearly separated, and individuals from IRS 9 were located between populations north and south of them. The largest genetic distance among *M. jonahi* individuals was inferred between populations from IRSs 12 to 14 and those north of them (clearly separated along PC1 in Figure S19), the latter of which stratified along PC2 as expected from their geographic location. This stratification and the clustering probabilities for IRS 9 aligned with the high rates of gene flow across IRSs 6–11 (Figure 2A, Table S8). Moreover, rates of gene flow from high‐ to lowland regions were generally higher than vice versa, and gene flow between highland regions was higher than that between lowland regions when considering the same adjacent IRSs. Notably, we also inferred gene flow between *M. macarthurii* and adjacent *M. jonahi* populations, which was further supported by a significant excess of shared alleles (Patterson's *D* = 0.26, *Z*‐score = 16.84, *p* < 0.0001; Figure S20).

In *M. lehilahytsara* , population structure was best explained by *K* = 2 (Figure S17), and populations in IRSs 5 and 6 as well as in the far north of the study region, which represent the highlands of IRS 4, showed partial assignment to both clusters (Figure 2B). Increasing *K* largely resulted in clusters corresponding to single IRSs (Figure S21). Clustering probabilities at *K* = 2 were consistent with the clinal pattern of genetic differentiation indicated by PCA (Figure S19). High levels of gene flow were found between populations throughout the entire region (Figure 2B, Table S9), including those on opposite sides of the large Antainambalana River. Similar to *M. jonahi* , gene flow from high‐ into lowland populations was on average higher than vice versa.

The population structure of *M. simmonsi* was characterised by the high differentiation between the northern populations in IRSs 11 and 11a (Île Ste. Marie) and the southern ones in IRSs 15–17, which was already evident in the phylogeny. These two clades corresponded to two distinct clusters in the clustering analysis (best *K* = 2; Figure 2C, Figures S17 and S22). In addition, the PCA supported high differentiation of the two clusters along PC1 (Figure S19), and gene flow was predominantly inferred within them (except for IRS 15 to IRS 11; Figure 2C, Table S10). Gene flow within the northern clade was inferred mainly from IRS 11a to IRS 11.

Finally, *K* = 2 was inferred as the best number of clusters for *A. laniger* and *A. mooreorum* (Figure S17), with each species corresponding to one cluster (Figure 2D, Figure S23). High rates of gene flow between neighbouring IRSs were inferred throughout the species' entire range in northeastern Madagascar (Figure 2D). This was also supported by principal component analysis, where adjacent IRSs clustered closely together (Figure S24). Moreover, the coalescent model inferred minor gene flow (Table S11) between *A. laniger* (IRS 5) and *A. mooreorum* (IRS 4), which was congruent with a slight but significant excess of shared alleles between *A. laniger* (IRS 5) and *A. mooreorum* (IRS 2/3 and 4; Patterson's *D* = 0.06, *Z*‐score = 4.22, *p* < 0.0001; Figure S25).

### 3.4 Drivers of Population Genetic Structure

**FIGURE 3:**
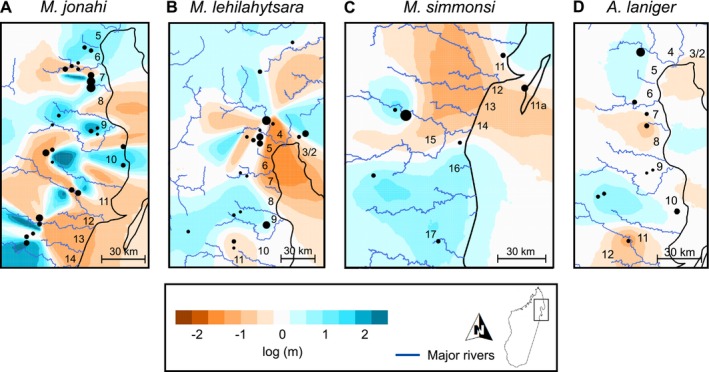
Estimated effective migration surfaces (EEMS) for the four species *M. jonahi* (A), *M. lehilahytsara* (B), *M. simmonsi* (C) and *A. laniger* (D) in northeastern Madagascar. Effective migration is displayed on a log_10_ scale. Numbers indicate inter‐river systems.

A significant pattern of IBD across the distributional range was found among individuals of all tested species ( *M. jonahi* : *r*_s_ = 0.88, *p* = 0.0001; *M. lehilahytsara* :*r*_s_ = 0.75, *p* = 0.0001; *M. simmonsi* : *r*_s_ = 0.81, *p* = 0.0001; *A. laniger* : *r*_s_ = 0.70, *p* = 0.0001; Figure S26). Population‐based tests were significant for *M. jonahi* (*r*_s_ = 0.82, *p* = 0.0001) and *M. lehilahytsara* (*r*_s_ = 0.58, *p* = 0.0002) but not for *M. simmonsi (r*_s_ = 0.39, *p* = 0.12) and *A. laniger* (*r*_s_ = 0.02, *p* = 0.45), potentially because of lower statistical power due to reduced sample size (Figure S27). Spatial deviations from a null model of IBD aligned with major rivers in *M. jonahi* (negative log migration), e.g., those separating IRSs 6 and 7, 9 and 10, 11 and 12, or 12 and 13 (Figure 3A). Log migration rates within IRSs were largely positive. In *M. lehilahytsara* , log migration rates were positive throughout much of the study region (Figure 3B). Negative estimates were found around the Antainambalana River, the rivers south of Marojejy NP (in the far north) and the coastal areas at the northern end of Antongil Bay. *M. simmonsi* showed negative log migration values between the northern clade (IRSs 11 and 11a) and the southern clade (IRSs 15–17) and, to a smaller extent, between populations in IRS 15 and those in IRSs 16 and 17 (Figure 3C). In *A. laniger* , negative log migration rates were found between IRSs 10/11 and 12 as well as around IRSs 7 and 8 (Figure 3D).

**FIGURE 4:**
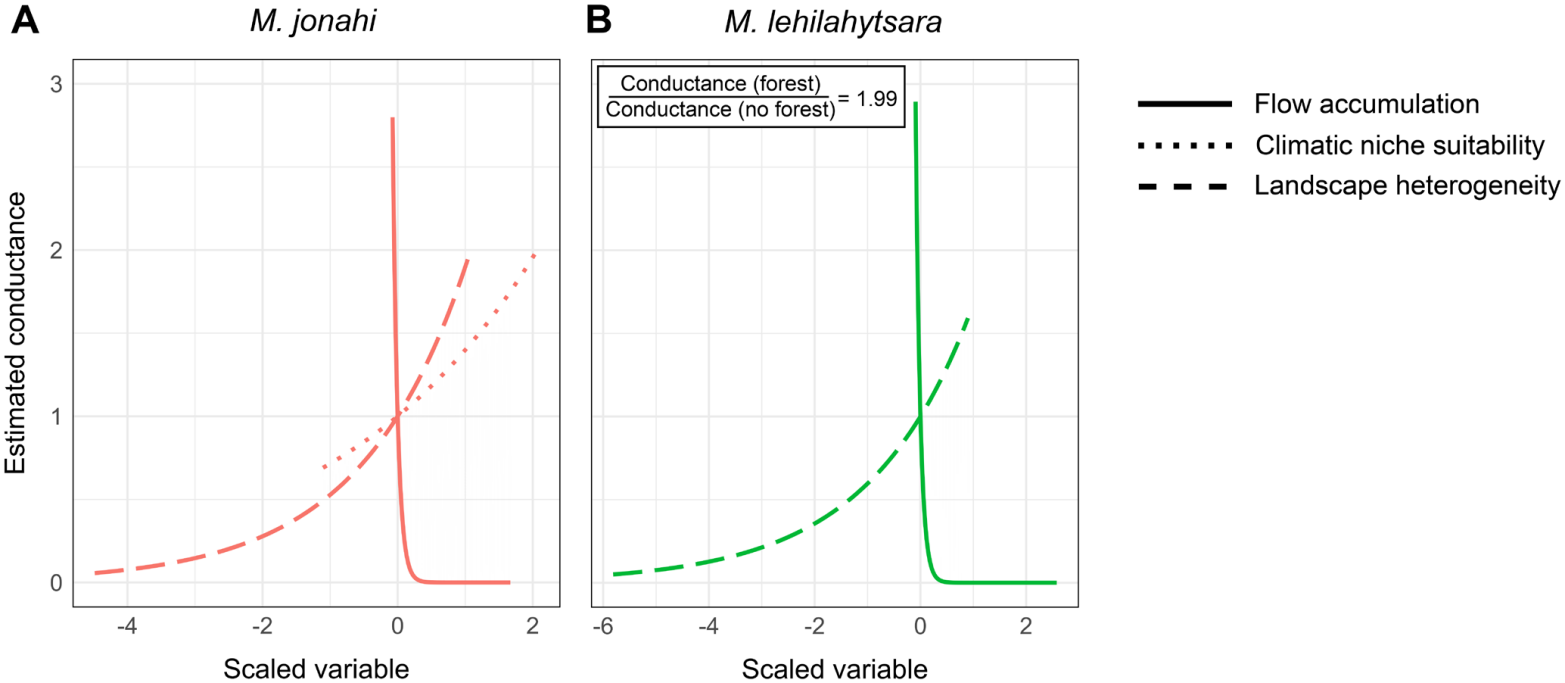
Estimated conductance associated with scaled values of predictor variables with significant effect in the best fitting models of isolation‐by‐resistance in *M. jonahi* (A; red) and *M. lehilahytsara* (B; green). The relative conductance of forest cover (categorical variable) in comparison to its absence is given in the inlet for *M. lehilahytsara* .

All univariate isolation‐by‐resistance models performed better (measured by AIC) than the null model of isolation‐by‐distance in *M. jonahi* and *M. lehilahytsara* (except for elevation in *M. lehilahytsara* ; Tables S4 and S5). In addition, multivariate models generally had a better fit than univariate models. The best fitting model in *M. jonahi* retained flow accumulation, climatic niche suitability and landscape heterogeneity as significant predictors (Figure 4A). While flow accumulation, that is river size, imposed a high negative effect on conductance, climatic niche suitability and landscape heterogeneity promoted conductance in *M. jonahi* . In *M. lehilahytsara* , flow accumulation (negative effect), landscape heterogeneity and forest cover (both with a positive effect) were significant predictors in the best fitting model (Figure 4B). Conversely, no model performed significantly better than the null model in *M. simmonsi* and *A. laniger* (i.e., the null model had a ΔAIC < 2 compared to the best alternative model; Tables S6 and S7), potentially because of a lack of statistical power as well.

### 3.5 Genetic Diversity

There were significant differences in *H*_O_ among species in the study region (Kruskal–Wallis test: *H* = 319.65, df = 8, *p* < 0.0001; Figure S28, Tables S12 and S13). Specifically, *H*_O_ was significantly higher in *Avahi* than in *Microcebus* species, except for *M. lehilahytsara* . Within the genus *Microcebus*, mean *H*_O_ was approximately three times higher in *M. lehilahytsara* than in the other species (Dunn's test: to *M. jonahi* : *z* = 11.44, *p* < 0.0001; to *M. macarthurii* : *z* = −9.10, *p* < 0.0001; to *M. simmonsi* : *z* = −11.63, *p* < 0.0001). In addition, *M. jonahi* had significantly higher *H*_O_ than *M. macarthurii* (*z* = −3.83, *p* = 0.005) and *M. simmonsi* (*z* = −4.37, *p* < 0.0001). No significant differences were found in *H*_O_ between *Avahi* species, probably due to limited sampling size. However, mean *H*_O_ of *A. laniger* was approximately 1.5 times higher than that of *A. mooreorum* .

The correlations between *H*_O_ and latitude, longitude and elevation were significant in *M. jonahi* and *M. simmonsi* (Figures S29 and S30), but not in *M. lehilahytsara* (Figure S31). In *M. jonahi* , *H*_O_ showed a strong positive correlation with latitude (higher *H*_O_ was found at more northern regions; *r*_s_ = 0.86, *p* < 0.0001). The relationships with respect to longitude (*r*_s_ = 0.48, *p* < 0.0001) and elevation (*r*_s_ = −0.20, *p* = 0.0083) were also significant but weaker. In contrast, in *M. simmonsi* , lower *H*_O_ was found at more northern and eastern sampling sites (*r*_s_ = −0.39, *p* = 0.0185 and *r*_s_ = −0.56, *p* = 0.0004 for latitude and longitude, respectively) and at higher elevations (*r*_s_ = 0.47, *p* = 0.004). This pattern was largely driven by the fact that the northern *M. simmonsi* clade (IRS 11 and 11a) exhibited the highest latitude and longitude and the lowest elevation and had lower mean *H*_O_ estimates than the southern clade. In *A. laniger* , *H*_O_ was only correlated significantly with latitude (*r*_s_ = 0.57, *p* = 0.0019; Figure S32). It is important to note that, due to the topographical nature of the study region, longitude and elevation were negatively correlated in all four species.

## 4 Discussion

We integrated extensive genomic and geographic data across a large region of northeastern Madagascar, divided by potential river barriers, to identify shared and species‐specific drivers of phylogeographic structure in four *Microcebus* and two co‐distributed *Avahi* species. Our findings highlight the value of jointly considering phylogeography, landscape features, species ecology and (paleo)climatic history to understand diversification processes and the associated role of river barriers (e.g., distinguishing vicariant from secondary‐contact scenarios). We discuss these aspects in detail below, paying special attention to how ecological differences and temporal landscape dynamics mediate the role of rivers as barriers.

### 4.1 Contrasting Species Responses to Riverine and Topographic Barriers

We found substantial differences in population structure across the studied species which can largely be explained by varying responses to landscape features. Specifically, we detected strong population structure in *M. jonahi* , with genetic clusters largely corresponding to different IRSs (Figure 2A). Congruently, effective migration surfaces (Figure 3A) and IBR models (Figure 4) revealed that rivers are important barriers to gene flow in this species. Migration corridors between IRSs are only given in headwater regions where rivers become small enough to be bridged by vegetation and crossed by arboreal animals (Goodman and Ganzhorn 2004; Wilmé et al. 2006). However, these routes seem to be constrained for *M. jonahi* given that climatic niche suitability was a significant predictor of connectivity (Figure 4) and declines at higher elevations (i.e., 800–1000 m a.s.l.) under current climatic conditions (Figures S7 and S12). Consistent with this, high rates of gene flow were predominantly found between IRSs where vegetation bridges across rivers exist today at these or lower elevations (IRSs 6–11; Figure 2A), whereas the highest differentiation (between populations in IRS 12–14 and those to the north) coincided with the Simianona River, which is bridged by vegetation only at ca. 1000 m a.s.l.

In contrast, *M. lehilahytsara* populations were characterised by high rates of gene flow between IRSs (Figure 2B) and high genetic diversity throughout the entire range of the species in northeastern Madagascar (Figure S31). Although rivers were also inferred as barriers in *M. lehilahytsara* (Figure 4B), gene flow among adjacent highland populations and from high‐ into lowland regions (Figure 2C) suggests that the species' connectivity and genetic diversity are likely maintained by extensive highland migration and river crossings at headwaters (Goodman and Ganzhorn 2004; Wilmé et al. 2006). The presence of *M. lehilahytsara* on the Masoala Peninsula (IRS 2), previously proposed as a distinct *Microcebus* lineage (*M.* sp. #2; Louis and Lei 2016), suggests the species can cross even large rivers such as the Antainambalana. This hypothesis is further supported by a lack of elevation and climatic niche as significant predictors in the best fitting IBR model (Table S5), indicating that *M. lehilahytsara* possesses substantial habitat flexibility (see also Andriambeloson et al. 2021; Schüßler, van Elst, et al. 2025). This is potentially due to physiological or behavioural adaptations to environmental challenges associated with higher elevations (e.g., the use of prolonged torpor to buffer against unfavourable environmental conditions; Blanco et al. 2017). Notably, we did not find *M. lehilahytsara* in lowland regions of IRSs 6–8, 10 and 11 (Figure S1). These absences could be the result of local extinctions in the remaining lowland forests during the LGM. The larger‐bodied lowland mouse lemurs could have survived the coldest and driest conditions in all IRSs by a stronger reliance on heterothermy, which may not have been possible for small‐bodied *M. lehilahytsara* (Schüßler, van Elst, et al. 2025). Subsequent recolonisation could have been impeded by niche competition with *M. jonahi* and *M. simmonsi* (Schüßler, van Elst, et al. 2025).

*Avahi laniger* was similarly characterised by weak population structure and high rates of gene flow throughout the sampled region (Figure 2D). This suggests that *A. laniger* also disperses effectively across river barriers around river headwaters (we consistently sighted *Avahi* individuals in headwater regions of IRSs 13–15 but were unable to collect samples at those sites). Although genomic data indicate that *A. laniger* is absent from IRSs 2/3 and 4 (unlike *M. lehilahytsara* ), the species' supposed presence in Anjanaharibe‐Sud SR (Lei et al. 2008) demonstrates its ability to disperse around the Antainambalana River. Its further expansion towards Masoala may have been precluded by interspecific competition with *A. mooreorum* . The exact distributional boundary of *A. laniger* and whether the two species interbreed remain open questions. Although we identified a significant excess of shared alleles between *A. mooreorum* and *A. laniger* in IRS 5 compared to those of IRS 10 (Figure S25), the signal was only weak and not supported by clustering analysis (Figure S23). In addition, biasing effects such as population structure and demographic history cannot be ruled out (Tournebize and Chikhi 2025). Congruent with the high connectivity among *A. laniger* populations, the species exhibited relatively high observed heterozygosities throughout its range, which were approximately 1.5‐ and 3‐fold higher than those in *A. mooreorum* and *A. occidentalis* , respectively (Figure S28, Table S13).

These findings confirm that rivers play a crucial role in restricting gene flow in lemurs in northeastern Madagascar but also show that modelling species‐specific responses to landscape and climatic variables is crucial to capturing their full impact on the diversification and distribution of different species. Although this context‐dependent effect of river barriers has been discussed before in other taxa across the tropics (e.g., Janiak et al. 2022; Kopuchian et al. 2020; Patton et al. 2000; Peres et al. 1996; Schüßler, Bremer, et al. 2025; Weir et al. 2015), studies modelling the effect of continuous landscape features such as topography on genetic differentiation alongside rivers are still rare today (but see Fonseca et al. 2024). This is particularly the case for the biota of Madagascar, where rivers and elevational gradients are considered key to species diversification (Goodman and Ganzhorn 2004; Mercier and Wilmé 2013; Wilmé et al. 2006). Process‐based analyses in different species are crucial for identifying the ecological variables that modulate the impact of geographic barrier effects and, ultimately, for assessing how proposed diversification models apply across the diverse taxa of the island. We showed that even between two species of a cryptic genus ( *M. jonahi* and *M. lehilahytsara* ) substantial differences exist in how such barriers affect diversification. Heterogeneous patterns of gene flow and/or species turnover along Madagascar's east coast have also been documented in amphibians, reptiles and additional lemurs, but the underlying causes remain to be identified (Boumans et al. 2007; Gehring et al. 2012; Goodman and Ganzhorn 2004; Schüßler, Bremer, et al. 2025; van Elst et al. 2023). As the entire east coast is divided by rivers posing potential barriers to gene flow and dispersal also in other taxa (Schüßler, Bremer, et al. 2025), the potential for highland migration is likely one of the most significant predictors of connectivity and range sizes of eastern rainforest taxa in Madagascar, mirroring findings in Amazonian river systems (e.g., Naka et al. 2012; Peres et al. 1996; Weir et al. 2015). Congruently, Schüßler, Bremer, et al. (2025) showed that the elevation of the river watershed is one of the most important predictors of species turnover in a range of terrestrial taxa in eastern Madagascar. Our findings also highlight the importance of interspecific competition in shaping species distributions, as several absence records could not be explained by climatic or geographic barriers alone. Future studies should therefore incorporate data on biotic interactions alongside niche models or detailed habitat characteristics to identify phylogeographic drivers.

### 4.2 Refugial Dynamics and Landscape Evolution Shape Divergence Processes

Identifying the role of rivers in diversification processes requires not only a species‐specific ecological perspective but also a temporal one, accounting for shifts in river courses, discharge and associated refugial habitats among others. According to coalescent models by Poelstra et al. (2021) and van Elst et al. (2025), the diversification of *Microcebus* species took place during the Quaternary glaciation cycles of the last few hundred thousand years. While river courses in the study region likely remained relatively stable through this period (Liu et al. 2024), pronounced sea‐level fluctuations (~120 m; Bintanja et al. 2005) and shifts in discharge and aridity influenced habitat dynamics (Burney et al. 2004; de Menocal 2004; Gasse and Van Campo 2001). These are therefore crucial for understanding the diversification of species in the region.

While our findings confirm the species level distinction of *M. jonahi* and *M. macarthurii* originally suggested by Poelstra et al. (2021) and Schüßler, Blanco, et al. (2020) (Discussion in Data S1), the divergence of these two species cannot be explained by landscape features included in our models. Specifically, the river separating their two ranges has low‐elevation headwaters with high habitat suitability for *M. jonahi* (Figure S7) and a continuous forest cover across both ranges (Figure S11). Potentially, the divergence of the two species occurred due to allopatric speciation in separate Pleistocene refugia during drier periods. The presence of *M. macarthurii* on Nosy Mangabe, which would have been connected to the mainland when sea levels were about 40 m lower (Schüßler 2025), suggests that during such periods the species must have occupied adjacent lowland areas to be able to retreat to the island as sea levels subsequently rose. This contrasts with the mountain refugia hypothesis, which has been proposed as a key mechanism of diversification in other small vertebrates and arthropods (Camacho et al. 2021; Everson et al. 2020; Wesener et al. 2011; Wollenberg et al. 2008), as it implies the presence of forested habitats as refugia at lower elevations. The geomorphology of the flat coastal plains with slow flowing surface water and high amounts of sediment accumulation may have fostered the formation of riparian wetlands (Noe and Hupp 2009), supported by the high discharge of the Antainambalana River (Schüßler 2025). Mangrove forests may have also provided suitable habitats, as they do for *M. gerpi* and other lemurs (e.g., Gardner 2016; Rakotondravony et al. 2023; Wuesthoff et al. 2021). Therefore, we hypothesise that populations in IRS 5 (today's *M. macarthurii* ) occupied lowland refugia (potentially near the Antainambalana River) during drier, cooler periods and got separated by an increasingly arid landscape from ancestral *M. jonahi* as the Voloina River ran dry due to its lower‐elevation headwaters and small size (Mercier and Wilmé 2013; Wilmé et al. 2006). Thus, this river appears to be a secondary contact barrier rather than a cause of vicariance (Naka and Pil 2020), maintaining the disjunct distributions of *M. jonahi* and *M. macarthurii* , possibly alongside interspecific competition for critical resources (as hypothesised for *M. jonahi* and *M. simmonsi* in IRS 11; Schüßler, van Elst, et al. 2025). A general scenario for the colonisation of the southern part of the study region by *M. jonahi* is given in Discussion in Data S1. Similar refugial dynamics may furthermore explain the divergence of *A. laniger* and *A. mooreorum* (Discussion in Data S1).

Changing sea levels also offer a plausible explanation for the disjunct distribution and high genetic differentiation between northern (IRSs 11 and 11a) and southern (IRSs 15–17) *M. simmonsi* populations (Figure 2C). Like *M. macarthurii* , the species occurs on an island (Île Ste. Marie) which was connected to the mainland during drier (glacial) periods. Extrapolating river courses via flow accumulation from a digital elevation model (Schüßler 2025) suggests that the rivers separating IRS 11 from the southern *M. simmonsi* populations converged into a single river when sea levels were about 50–100 m lower than today (e.g., during the LGM; Figure S33). Accordingly, only one dispersal event across this river (e.g., through rafting or meander cutoff; Haffer 2008) would have been necessary to colonise Île Ste. Marie from IRS 16 and, subsequently, IRS 11 (Figure S33). The species' climatic niche (Figure S9) and a high connectivity across river barriers among southern populations (Figure 2C) indicate that *M. simmonsi* has a relatively high elevational tolerance. For instance, gene flow between the highlands of IRS 15 and IRS 16 may be mediated by dispersal through the marshes of Lac Alaotra, where mouse lemurs have already been reported even though their species identity remains to be confirmed (Garbutt 2022). Therefore, the absence of *M. simmonsi* in the highlands of IRS 11 is unexpected. Following its arrival, the species may not have been able to further expand its range due to competition with *M. jonahi* or larger‐bodied lemur species that already inhabited IRSs 11–14 (Schüßler, van Elst, et al. 2025; Thorén et al. 2011).

Together, these scenarios corroborate the need to account for Madagascar's dynamic landscape evolution – including shifts in sea level, river discharge and habitat connectivity—when interpreting the diversification of Malagasy biota (Clementucci et al. 2025; Liu et al. 2024; Mercier and Wilmé 2013; Wilmé et al. 2006). Adopting such a perspective is essential to distinguish primary barriers that have driven lineage divergence from those that now limit secondary contact (Naka and Pil 2020), such as the Voloina River. Moreover, our findings indicate that lowland regions along Madagascar's east coast may have functioned as important refugia driving divergence in certain taxa, complementing the prevailing emphasis on montane regions as centres of speciation in Madagascar (e.g., Camacho et al. 2021; Everson et al. 2020; Raxworthy and Nussbaum 1995). Similar dynamics have been shown in the Amazon and Southeast Asia, where rivers, coastal plains and/or transient land connections alternately promoted isolation and connectivity (Driller et al. 2015; Musher et al. 2022). Importantly, the diversification processes presented here are based on the interpretation of current distributions and patterns of genetic diversity and differentiation in light of paleoclimatic shifts. Explicit demographic models testing them against alternative explanations will be necessary to strengthen their support (e.g., Teixeira, Montade, et al. 2021).

### 4.3 Conservation Implications

Our comparative investigation of population structure and its determinants provides comprehensive information on the genetic diversity and gene flow of *Microcebus* and *Avahi* species in northeastern Madagascar, identifies promising targets for conservation (detailed conservation recommendations for each species are given in Discussion in Data S1). Despite interspecific differences in connectivity across inter‐river systems, all studied species except the microendemic *M. macarthurii* depend on highland and headwater migration corridors to maintain gene flow, which may apply to a wider range of taxa along Madagascar's east coast (Gehring et al. 2012; Goodman and Ganzhorn 2004; Schüßler, Bremer, et al. 2025). Accordingly, the protection of forest habitat around headwaters, where vegetation (e.g., the canopy) allows species to cross rivers, should be prioritised in conservation efforts. This is particularly relevant considering potential scenarios of climate change and associated migratory movements of species to track changing habitats, which are predicted to become prominent in northeastern Madagascar (Brown and Yoder 2015).

We also found a positive relationship of topographic complexity (i.e., landscape heterogeneity) and genetic connectivity in *M. jonahi* and *M. lehilahytsara* (Figure 4). Topographically complex regions may offer a range of relatively stable microclimates due to protective sheltering effects in connected habitat pockets, that may be used as migratory corridors. These advantageous ecological settings may facilitate the buffering of diurnal, seasonal and unpredictable interannual and historic climatic fluctuations (e.g., in temperature or humidity; Byrne et al. 2022; John et al. 2024). Such buffering effects are expected to be particularly relevant in Madagascar given its highly seasonal climate (Goodman 2022), pronounced variation in climatic conditions (Dewar and Richard 2007) and recurring natural events like cyclones (Donque 1975) and droughts (e.g., Gould et al. 1999; Virah‐Sawmy et al. 2010), the latter of which can also severely impact the humid rainforests of the island (Ranomafana NP, unpubl. records). It has already been suggested that these factors play a role in explaining the idiosyncratic life histories of lemurs compared to other primates (Dewar and Richard 2007; Wright 1999). Accordingly, topographically complex regions may serve as refugia for mouse lemurs and other taxa, potentially promoting higher population sizes, higher genetic diversity and dispersal, which makes them potential conservation targets. Such regions may already be associated with less human pressure (e.g., habitat loss and fragmentation) as they should be more difficult to access and cultivate (Schüßler, Mantilla‐Contreras, et al. 2020). Protecting refugia and dispersal corridors will be key to conserving lemur diversity in Madagascar's remaining humid forests amid the ongoing biodiversity crisis.

## Author Contributions

**Tobias van Elst:** conceptualisation (equal); data curation (lead); formal analysis (lead); funding acquisition (supporting); investigation (lead); methodology (lead); project administration (equal); software (lead); resources (equal); validation (lead); visualisation (lead); writing – original draft (lead); writing – review and editing (lead). **Dominik Schüßler:** conceptualisation (supporting); data curation (supporting); funding acquisition (supporting); project administration (equal); resources (equal); visualisation (supporting); writing – original draft (supporting); writing – review and editing (supporting). **Stephan M. Rafamantanantsoa:** project administration (supporting); resources (supporting). **Tahiriniaina Radriarimanga:** project administration (supporting); resources (supporting). **Naina R. Rabemananjara:** project administration (supporting); resources (supporting). **David W. Rasolofoson:** project administration (supporting); resources (supporting). **R. Doménico Randimbiharinirina:** project administration (supporting); resources (supporting). **Paul A. Hohenlohe:** investigation (supporting); methodology (supporting); resources (supporting). **Ute Radespiel:** conceptualisation (equal); data curation (supporting); funding acquisition (lead); investigation (supporting); methodology (supporting); project administration (equal); resources (equal); supervision (lead); validation (supporting); writing – review and editing (supporting).

## Funding

This project was funded by the German Research Foundation (DFG Ra 502/23‐1 to U. R.), the Society for Tropical Ecology, Houston Zoo Inc., the Bauer‐Hollmann Foundation (T237/22985/2012/kg to D. S.), the Zemplin Foundation (T0214/32083/2018/sm to D. S.) and through a compute project provided by the German National High Performance Computing Alliance NHR@Göttingen (nib00015 to U. R. and T. v. E.).

## Disclosure

Benefit‐Sharing: Our group is committed to international scientific partnerships, as well as institutional capacity building in Madagascar. Accordingly, this research was conducted in close collaboration with Malagasy universities and included outreach and training of local students. In addition, the field work of this research was conducted through the help of local communities in the study region, which were employed as guides, cooks and porters. All field and scientific procedures adhered to Malagasy regulations, the Nagoya Protocol, standards of the International Primatological Society (International Primatological Society 2014) and the ‘proposal for ethical research conduct in Madagascar’ (Wilmé et al. 2016). All collaborators that contributed to the scientific development of this project are included as co‐authors. Specifically, five of nine co‐authors are Malagasy. The results of this collaboration have been shared with local authorities given that the research has direct implications for the conservation of Madagascar's biodiversity.

## Conflicts of Interest

The authors declare no conflicts of interest.

## Supporting information


**Data S1:** mec70195‐sup‐0001‐DataS1.zip.

## Data Availability

All new sequencing data have been made available through NCBI BioProject PRJNA807164. Individual BioSample accessions are given in Table S1. VCF files and analysis outputs are available at Dryad (https://doi.org/10.5061/dryad.4xgxd25nt). Scripts can be found at https://github.com/t‐vane/ResearchSupplements.
