# Supplementary material for: Species‐Specific Responses to Paleoclimatic Changes and Landscape Barriers Drive Contrasting Phylogeography of Co‐Distributed Lemur Species in Northeastern Madagascar
Source: Mol Ecol. 2025 Dec 10;34(24):e70195. doi: 10.1111/mec.70195 (PMC12717996; doi:10.1111/mec.70195)
Supplement: Supplementary file 1 — Data S1: mec70195‐sup‐0001‐DataS1.zip. [file MEC-34-e70195-s001.zip › mec70195-sup-0001-Supinfo.pdf]

**Supplementary material**

# Species-specific responses to paleoclimatic changes and landscape barriers drive contrasting phylogeography of co-distributed lemur species in northeastern Madagascar

Tobias van Elst[1,2], Dominik Schüßler[3], Stephan M. Rafamantanantsoa[4], Tahiriniaina Radriarimanga[4,5], Naina R. Rabemananjara[1,4,5], David W. Rasolofoson[5], R. Doménico Randimbiharinirina[5], Paul A. Hohenlohe[6], Ute Radespiel[1,*]

[1] Institute of Zoology, University of Veterinary Medicine Hannover, Hanover, Germany
[2] Animal Genomics, ETH Zurich, Zurich, Switzerland
[3] Institute of Biology and Chemistry, University of Hildesheim, Hildesheim, Germany
[4] Mention Anthropobiologie et Développement Durable, Faculté des Sciences, Université d'Antananarivo, Antananarivo, Madagascar
[5] Groupe d'Étude et de Recherche sur les Primates de Madagascar (GERP), Antananarivo, Madagascar
[6] Department of Biological Sciences, University of Idaho, Moscow, Idaho, USA

* Corresponding author: ute.radespiel@tiho-hannover.de

## 1 Supplementary methods

### 1.1 Restriction-site associated DNA sequencing (RADseq)

RADseq libraries were generated with the *SbfI* restriction enzyme following three different approaches (detailed for each sample in Table S1):

(1) Libraries were prepared according to the protocol described in Ali et al. (2016) at the University of Idaho and sequenced on an Illumina HiSeq 4000 (paired-end 150 bp) at the Vincent J. Coates Genomic Sequencing Laboratory of the University of California Berkeley, USA.

(2) Libraries were prepared according to the protocol described in Genomic Resources Development Consortium et al. (2015) and sequenced on an Illumina HiSeq 2000 (single-end, 100 bp) at the University of Oregon Core Facility, USA.

(3) Library preparation and sequencing were performed with the following custom protocol at Biosearch Technologies (LGC Genomics GmbH, Berlin, Germany). 50 ng of genomic DNA were digested with *SbfI* in 12 µl CutSmart® buffer (New England Biolabs; NEB) for 30 min at 37 °C. Restriction digests were mixed with 1.5 µl of barcoded forward *PstI* adaptors and 20 µl ligation master mix (containing 15 µl and 0.4 µl NEB Quick Ligation™ buffer and ligase, respectively). Ligation reactions were incubated for 35 min at 25°C (first ligation step). Subsequently, reactions were diluted with 30 µl Tris buffer, mixed with 50 µl Agencourt XP beads (Beckman Coulter), incubated for 15 min at room temperature, and placed for 5 min on a magnet to collect the beads. The supernatant was discarded, and the beads were washed twice with 200 µl 80% ethanol. Beads were air dried for 10 min and purified DNA was eluted in 10 µl Tris Buffer (first purification step). Subsequently, 7.5 µl of purified DNA was mixed with 7.5 µl of a master mix containing 70% Fragmentase buffer and 30% Fragmentase enzyme (Allegro® Genotyping Kit, Tecan/NuGen). Reactions were incubated for 20 min at 25°C (fragmentation/end repair step). Reaction products were again mixed with 30 µl ligation master mix (containing 22.5 µl and 0.6 µl NEB Quick Ligation™ buffer and ligase, respectively, and 10 pM standard blunt reverse adaptors). Ligation reactions were incubated for 35 min at 25°C (second ligation step). Reactions were then diluted with 20 µl Tris buffer, mixed with 50 µl Agencourt XP beads (Beckman Coulter), incubated for 15 min at room temperature, and placed for 5 min on a magnet to collect the beads. The supernatant was discarded, and the beads were washed twice with 200 µl 80% ethanol. Beads were air dried for 10 min and purified DNA was eluted in 10 µl Tris buffer (second purification step). Libraries were amplified during 19 cycles in 20 µl PCR reactions using MyTaq™ polymerase and mix (Bioline) and standard TruSeq amplification primers (Illumina). Finally, 5 µl from each of 96 amplified libraries were pooled. PCR primer and small amplicons were removed by Agencourt XP bead purification using one volume of beads. The PCR enzyme was removed by an additional purification on MinElute Columns (Qiagen). The pooled library was eluted in a final volume of 20 µl Tris buffer, size-selected on an LMP agarose gel (removing fragments smaller than 300 bp and larger than 650 bp) and sequenced on an Illumina NovaSeq 6000 (paired-end, 150 bp).

### 1.2 Genotype filtering

Genotype sets were filtered following FS6 filtering recommendations of O'Leary et al. (2018) with modified thresholds. Unless otherwise mentioned, the filtering was done in VCFtools v0.1.17 (Danecek et al., 2011) as follows:

(1) Genotypes with a per-sample depth less than 5 were masked.

(2) Monomorphic sites and those with a mean depth across samples less than 15 were removed.

(3) Three rounds of filtering sites and individuals based on missing data where applied, with the following thresholds for the maximum percentage of missing data:

(a) Site: 50%; Individual: 90%
(b) Site: 40%; Individual: 70%
(c) Site: 30%; Individual: 50%

(4) Sites were subsequently annotated with INFO fields for strandedness of reference vs. alternative allele (FS), root mean square mapping quality (MQ), mapping quality of reference vs. alternative allele (MQRankSum), read position of reference vs. alternative allele (ReadPosRankSum) and allele balance (ABHet), using GATK v3.8.1 (McKenna et al., 2010). Sites meeting one of the following criteria were removed:

(a) FS > 60.0
(b) MQ < 40.0
(c) MQRankSum < −12.5
(d) ReadPosRankSum < −8.0
(e) ABHet < 0.2 or ABHet > 0.8

This filter was only applied for the set of *Microcebus* samples because genotypes in *Avahi* samples were called using a reference-free approach in Stacks v2.53 (Rochette et al., 2019).

(5) Sites with a mean depth across samples larger than the mean depth across all sites plus twice the standard deviation were removed.

(6) Sites with more than 10% and individuals with more than 25 % of missing data were removed as in step (3).

(7) Sites with a minor allele count less than 3 were removed.

### 1.3 Drivers of population genetic structure

The following samples were not considered for isolation-by-resistance modeling (see also Table S1):

(1) *M. lehilahytsara* individuals south of the study region, as we focused on landscape effects in the focal region and wanted to avoid introducing large unsampled areas, which may bias model inference.

(2) *M. simmonsi* individuals from IRS 11 and 11a (Ambodiriana and Île Ste. Marie), as the large distributional gap between these populations and those in IRSs 15–17 suggests that the associated high genetic distances are not explained by continuous landscape variables.

(3) *M. macarthurii* and *A. mooreorum* individuals, as potential reproductive barriers to their sister species may bias model inference. Sample size did not allow modeling these species separately.

Climatic niche models were based on the bioclimatic variables isothermality, temperature seasonality, maximum temperature of warmest month, minimum temperature of coldest month, annual precipitation, precipitation seasonality, precipitation of wettest and driest quarter, which were previously shown to be ecologically relevant to mouse lemurs (Kamilar et al., 2016; Karger et al., 2017). All eight variables were subjected to PCA, and only the first three PCs were selected (together explaining 93.1% of the variation) to reduce multicollinearity and control for low sample sizes. The MaxEnt-algorithm of the R package 'ENMtools' v1.1.2 (Warren et al., 2021) was then used for climatic niche model estimation, and parameters were tuned independently based on lowest AIC value, using 10,000 background points. Model validation was based on the area under the receiver operating curve (AUC) and the continuous Boyce index (CBI) with a fivefold cross validation approach in the R package 'ENMeval' v2.0.4 (Kass et al., 2021). We did not test the role of past climatic niche suitability in explaining genetic distances (e.g., as done in Fonseca et al., 2024) because paleoclimatic models for Madagascar are associated with high uncertainty (Sordyl, 2022).

## 2 Supplementary results

### 2.1 Species distributions

*M. jonahi* was previously only known from IRSs 9 and 10 (Schüßler et al., 2020; Poelstra et al., 2021) but was now found at all sampled sites between the Voloina River in the north and the Sandratsio River in the south (IRSs 6–14) and at elevations from 40–830 m a.s.l. (Fig. S1; Table S1). Interestingly, unlike hypothesized in van Elst et al. (2025), its sister species *M. macarthurii* seems to be confined to IRS 5, as it was not found anywhere else.

The widely distributed *M. lehilahytsara* had so far been reported from several highland sites in the northeast as well as from lower elevations at Anjiahely (formerly *M. mittermeieri*; IRS 5) and Ambavala (IRS 9; see Poelstra et al. 2021; Tiley et al. 2022), where it occurs sympatrically with *M. macarthurii* and *M. jonahi*, respectively. We found *M. lehilahytsara* individuals at most sampling sites between the Masoala peninsula and the Simianona River (IRSs 2/3–11; Fig. 2B; Table S1). However, the species does not seem to occur regularly in low-elevation regions that are also inhabited by *M. jonahi* (e.g., in IRSs 6–8, 10), except for Ambavala. In contrast, *M. lehilahytsara* was found at lowland sites in IRS 2/3 and 4, where no other *Microcebus* species was observed.

*M. simmonsi* was reported from lowland forests in IRS 11 (Ambodiriana), Île Ste. Marie (formerly *M. boraha*; IRS 11a), and three sites south of the Maningory River (IRSs 16 and 17) prior to this study (Andriamasimanana et al., 2001; Rakotondravony & Rabenandrasana, 2011; Raxworthy, 1986;

Schüßler et al., 2020). Our new sampling adds one occurrence record in IRS 15 (Befotaka) to this distribution (Fig. 2C; Table S1). Notably, we did not find any *M. simmonsi* individuals between the Simianona and Sandratsio Rivers despite sampling all three interjacent IRSs (12–14), which contained *M. jonahi* instead. This suggests a distributional gap between the northern and southern sampling sites of *M. simmonsi*.

Finally, the Antainambalana River presents a distributional boundary towards the Masoala peninsula for *A. laniger* (Figs. 2D, S2; Table S1), which has been assumed to occur along Madagascar's east coast between the Mangoro River in the south and the Bemarivo River in the north (Mittermeier et al., 2023). Mitochondrial data by Lei et al. (2008) indicate that the distribution of *A. laniger* extends north up to Anjanaharibe-Sud Special Reserve, but our sampling did not allow testing this hypothesis (Fig. S2). We show, however, that IRS 4 is occupied by *A. mooreorum* instead (Figs. 2D, S2; Table S1), previously only known around its type locality in IRS 2/3 (Lei et al., 2008; Mittermeier et al., 2023).

## 3 Supplementary discussion

### 3.1 Species delimitation of *M. jonahi* and *M. macarthurii*

While Poelstra et al. (2021) detected genetic differentiation between *M. jonahi* and *M. macarthurii*, they could not exclude the possibility that sampled populations represented opposite ends of a cline in genetic variation due to a lack of data for IRSs 6–8. We filled this sampling gap and showed that the two species are reciprocally monophyletic (Fig. 1A), with high genetic distances (even at small geographic distances; Figs. S26, S27) and significant differences in genetic diversity (Fig. S28, Table S13). Accordingly, our findings confirm the species level distinction of the two taxa originally suggested by Poelstra et al. (2021) and Schüßler, Blanco, et al. (2020). Like several other *Microcebus* species (e.g., *M. berthae, M. sambiranensis*; see van Elst et al., 2025), *M. macarthurii* seems to be an extreme microendemic with a restricted range in the lowland forests of IRS 5 and on the island of Nosy Mangabe (not sampled; Louis & Lei, 2016), explaining its reduced genetic diversity. Notably, we inferred low levels of gene flow between the two species (Fig. 2A), supported by low probabilities of *M. jonahi* individuals in IRS 6 to belong to *M. macarthurii* clusters ($K = 3$ and $K = 4$) and a significant excess of shared alleles compared to more southern *M. jonahi* populations (Figs. S18, S20). This mirrors previous findings of mitochondrial introgression from *M. jonahi* into *M. macarthurii* by Poelstra et al. (2021) and Radespiel et al. (2008). However, it is important to note that factors such as population structure, demographic history, and gene flow from ghost lineages may generate an excess of shared alleles in addition to or instead of introgression (e.g., Martin et al., 2014; Tricou et al., 2022; Tournebize & Chikhi, 2025). For instance, a structured model in which the focal taxa derive from a common ancestral population, a plausible scenario for *M. jonahi* (IRS 6) and *M. macarthurii*, can produce spurious introgression signals (Tournebize & Chikhi, 2025). Estimates of the $D$-statistic can also be inflated by

small effective population sizes, e.g. because of population bottlenecks (Martin et al., 2014), which may have occurred during the demographic history of *M. macarthurii* considering its lower genetic diversity (Fig. S29) and restricted range (Fig. S1). Therefore, the processes underlying the inferred sharing of alleles should be confirmed through explicit demographic models.

### 3.2 Colonization scenario for *M. jonahi*

According to coalescent models by Poelstra et al. (2021) and van Elst et al. (2025), the diversification of *M. jonahi* and *M. macarthurii* took place during the Quaternary glaciation cycles of the past 200,000 years. We do not know, however, whether the colonization of the study region by *M. jonahi* started already before, during or after its divergence from *M. macarthurii*. In any case, the gradual decline in genetic diversity towards the southern end of *M. jonahi*'s range (Fig. S29) suggests that its ancestor colonized the region from a central northern region (potentially a refugium). While either the range of *M. macarthurii* or the Antainambalana River, with its comparably high-elevation source at ca. 1,230 m a.s.l. (Schüßler et al., 2025), likely served as a northern dispersal barrier, colonization towards the south may have been facilitated by the relatively lower headwater elevations of rivers separating IRSs 6–11. The initial colonization of IRS 12–14 despite the high-elevation headwaters of the Simianona and Marimbona Rivers may be explained by more favorable (warmer) climatic conditions during past interglacial periods, possibly allowing *M. jonahi* to disperse via higher elevations (Wilmé et al., 2006). Potentially, historical highland populations of *M. jonahi* may have subsequently lost favorable habitats with an increasing aridification during the LGM or have been outcompeted by the newly arriving *M. lehilahytsara* following its south-to-north colonization of the same region ca. 100 ka ago (Tiley et al., 2022). However, given that *M. jonahi* is significantly larger than *M. lehilahytsara* (Schüßler et al., 2023) and that *M. lehilahytsara* seems to shift its ecological niche when living in sympatry with *M. jonahi* (Schüßler, van Elst, et al., 2025), the latter explanation is not very likely.

### 3.3 Divergence of *A. laniger* and *A. mooreorum*

Even though direct divergence time estimates are not available for *A. laniger* and *A. mooreorum*, Herrera & Dávalos (2016) indicated that the diversification of the genera *Microcebus* and *Avahi* in eastern Madagascar occurred on a similar time scale. Therefore, the paleoclimatic dynamics that affected mouse lemurs may have also shaped the diversification of *Avahi* species. Specifically, we hypothesize that the ancestor of the two sister species studied here may have become separated in different refugia alongside the Antainambalana River during more arid conditions. Ancestral *A. laniger* may have retreated to a high-elevation refugium or to the southern side of the Antainambalana River while another part of the ancestral population (evolving into *A. mooreorum*) remained confined to the

Masoala peninsula, eventually leading to their divergence. The subsequent recolonization of the study region by *A. laniger* and its dispersal along the Antainambalana River would have led to the current distribution patterns. The fact that the distribution of *A. mooreorum* remained relatively restricted could also be due to competition with its sister species, as hypothesized for *M. macarthurii* and *M. jonahi* in IRS 5 and *M. simmonsi* and *M. jonahi* in IRS 11. As observed heterozygosities were similarly high across the entire range of *A. laniger* (likely due to its high connectivity; Fig. S32), our data do not allow us to identify a specific refugium and colonization route.

### 3.4 Conservation recommendations

As all studied species depend on highland and headwater migration corridors to maintain genetic connectivity, the protection of forest habitat around headwaters, where vegetation allows species to cross rivers, should be prioritized in conservation efforts. The extensive Makira Natural Park already protects headwater regions in the northern part of the distribution of *M. jonahi* (extending south to IRS 7), but its southern half, which harbors populations with the lowest genetic diversity (Fig. S29), and potential migration corridors remain largely unprotected (except for Mananara-Nord NP in the lowlands of IRS 10 and Ambatovaky SR in the highlands of IRS 12; Ralimanana et al., 2022). Particular attention should also be given to an effective protection of lowland forest habitats of IRS 5 (Anjiahely) and the island of Nosy Mangabe as these seem to be the only regions harboring the microendemic *M. macarthurii*, which already shows low genetic diversity compared to the other studied *Microcebus* species (currently protected as Makira Natural Park and Nosy Mangabe NP). The isolated *M. simmonsi* populations in IRS 11 (Ambodiriana) and on Île Ste. Marie (IRS 11a) also deserve urgent conservation attention, as to date, no formal protection status has been given to these sites (Ralimanana et al., 2022).

## 4 Supplementary figures

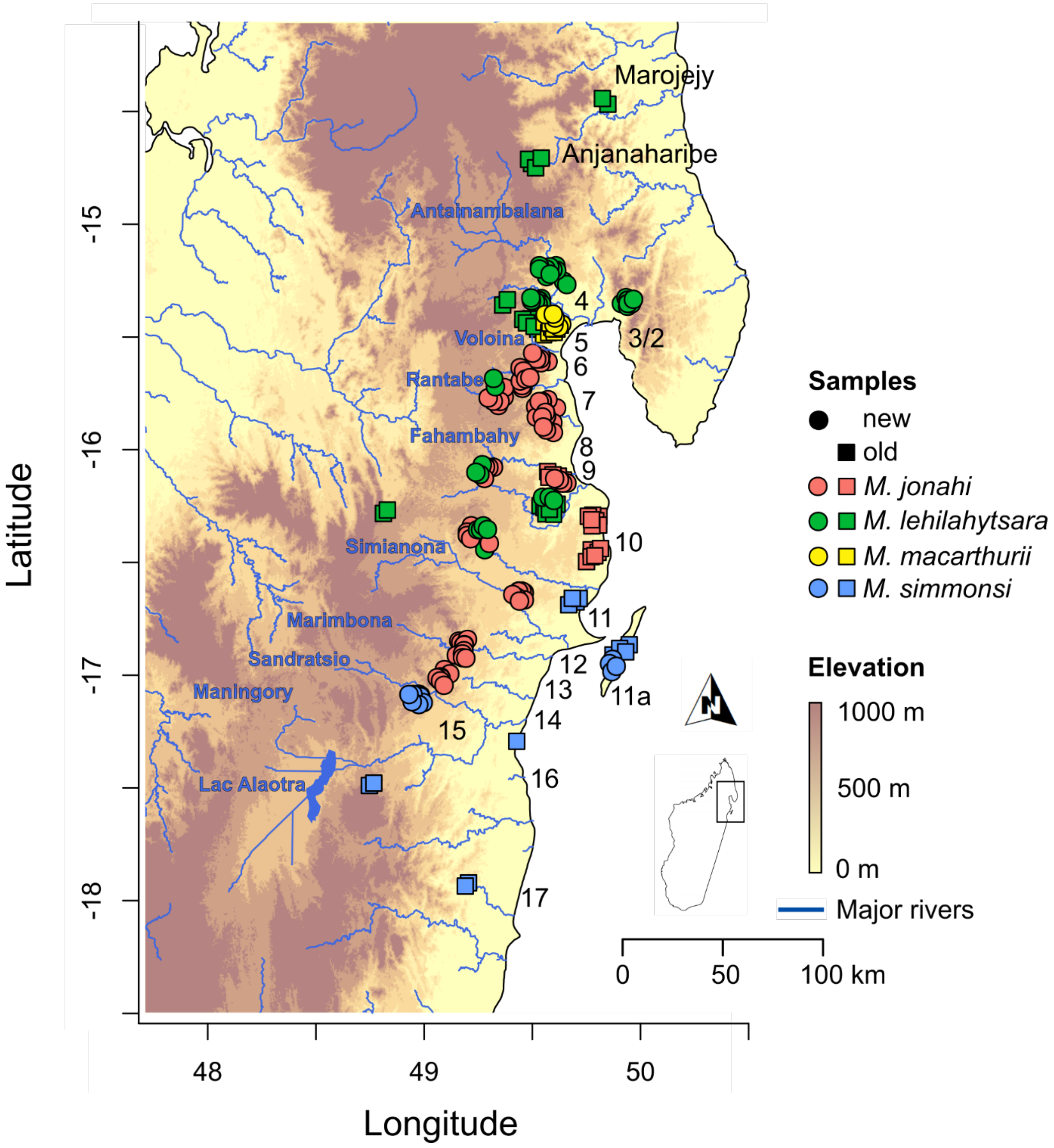


**Fig. S1:** Sampled *Microcebus* spp. individuals across the study region in northeastern Madagascar. Sample information is given in Table S1. Numbers denote inter-river systems. Blue labels denote names of rivers and Lac Alaotra.

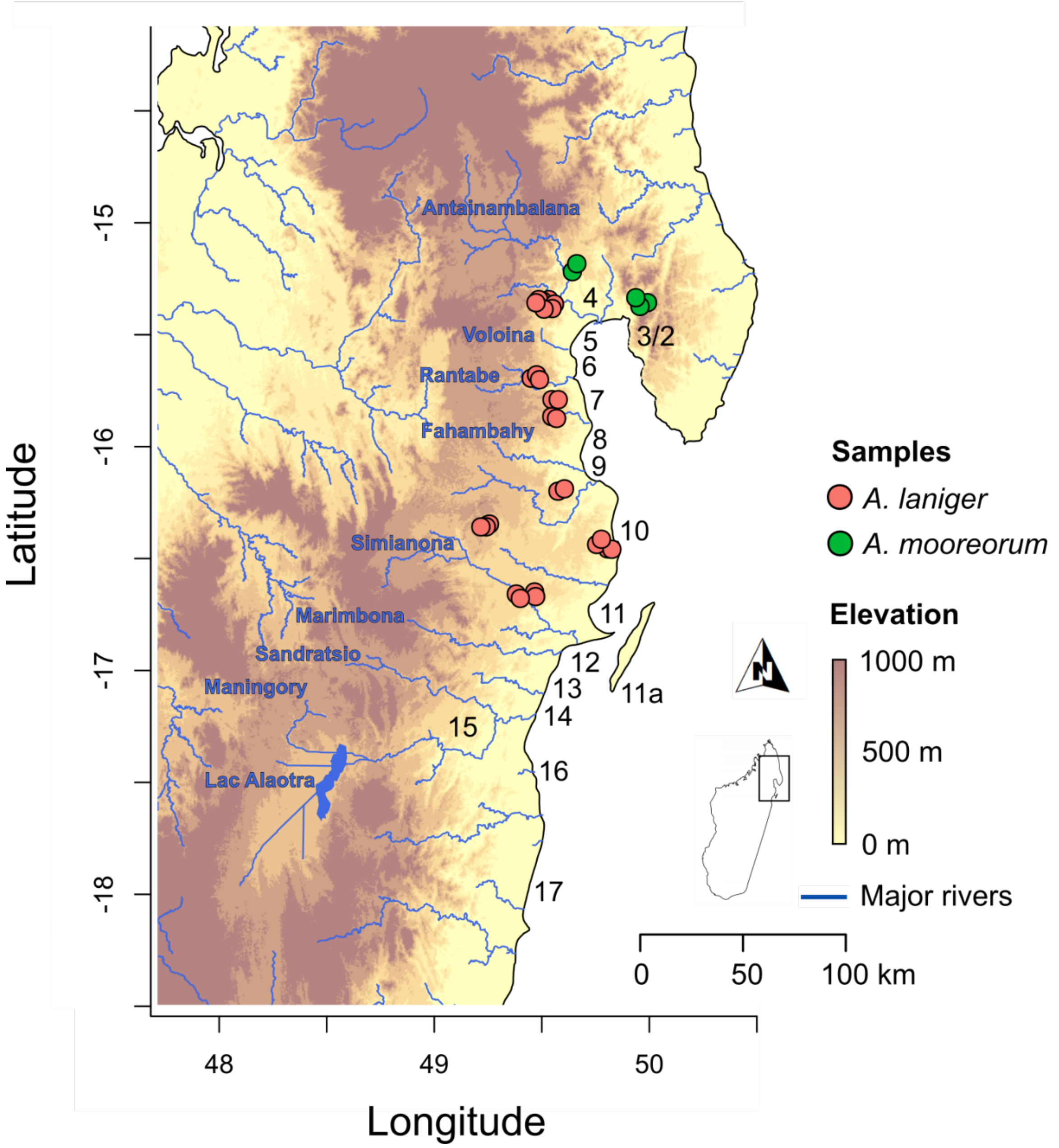


**Fig. S2:** Sampled *Avahi* spp. individuals across the study region in northeastern Madagascar. Sample information is given in Table S1. Numbers denote inter-river systems. Blue labels denote names of rivers and Lac Alaotra.

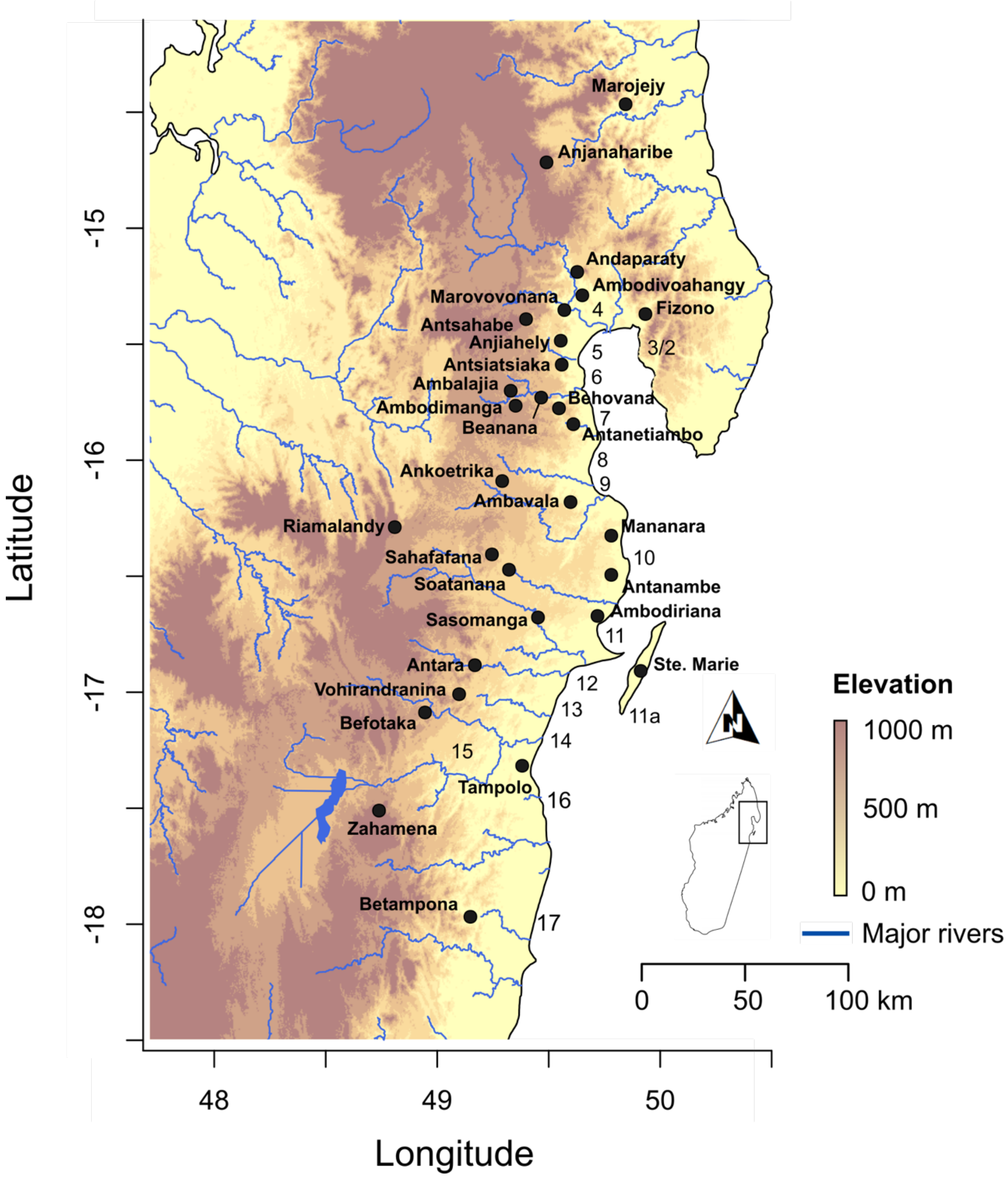


**Fig. S3:** Names of sampling sites in the study region in northeastern Madagascar. Sample information is given in Table S1. Numbers denote inter-river systems.

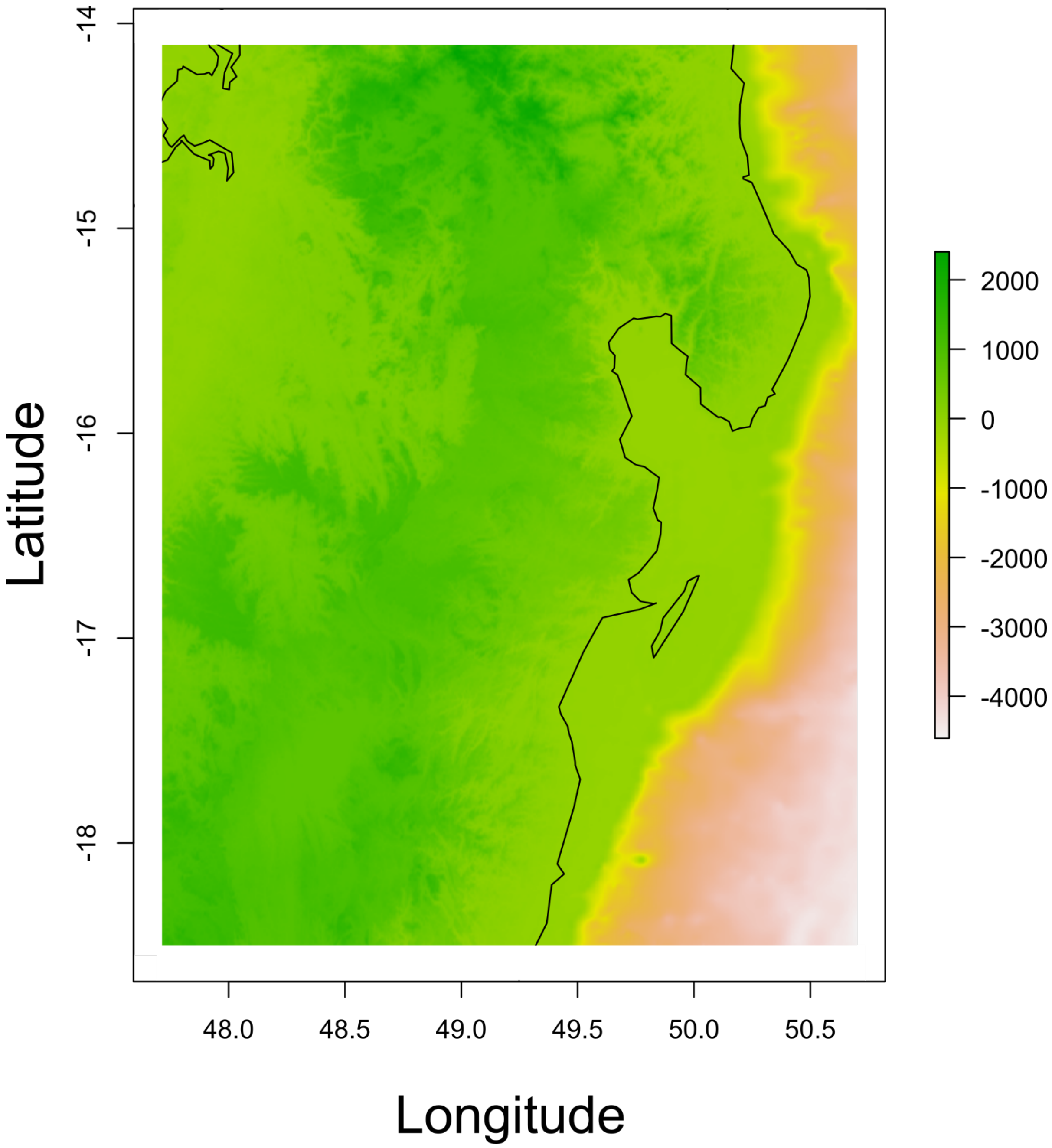


**Fig. S4:** Elevation (m) raster across the entire study region (not scaled) used for isolation-by-resistance analysis. Resolution is 150 m per pixel.

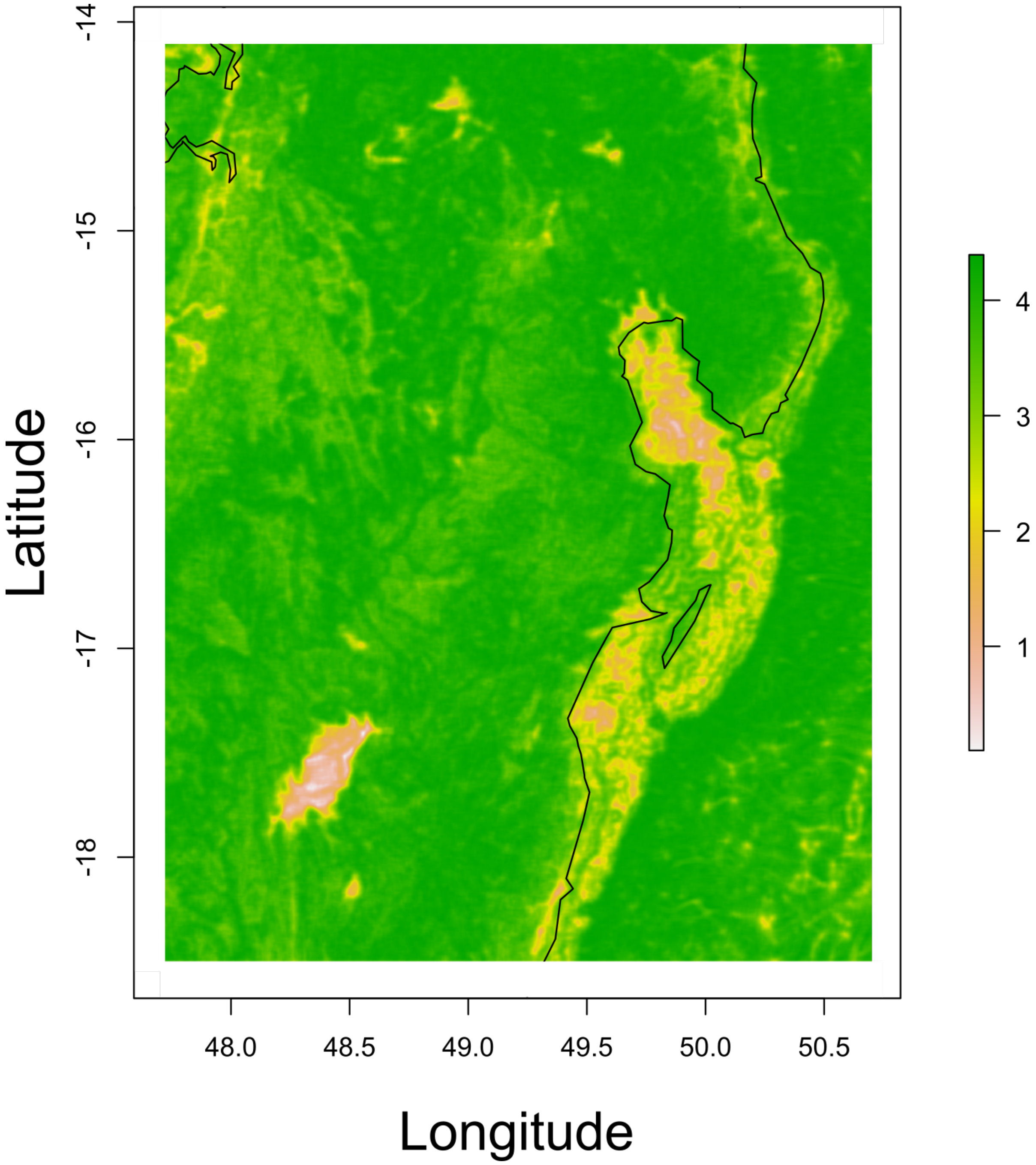


**Fig. S5:** Landscape heterogeneity raster across the entire study region (not scaled) used for isolation-by-resistance analysis. Resolution is 150 m per pixel.

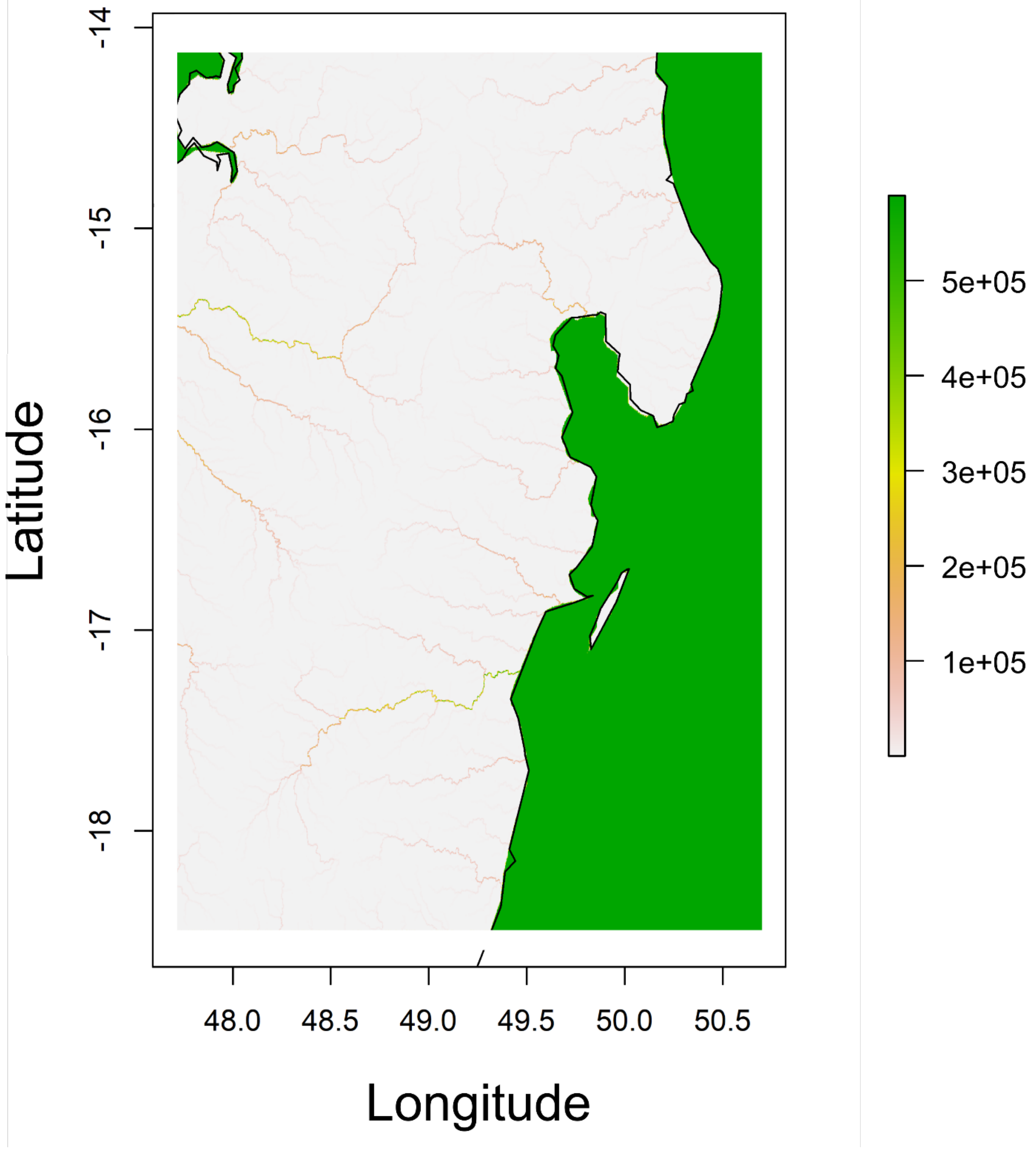


**Fig. S6:** Flow accumulation raster across the entire study region (current sea level; not scaled) used for isolation-by-resistance analysis. Resolution is 150 m per pixel. River width was increased for visibility.

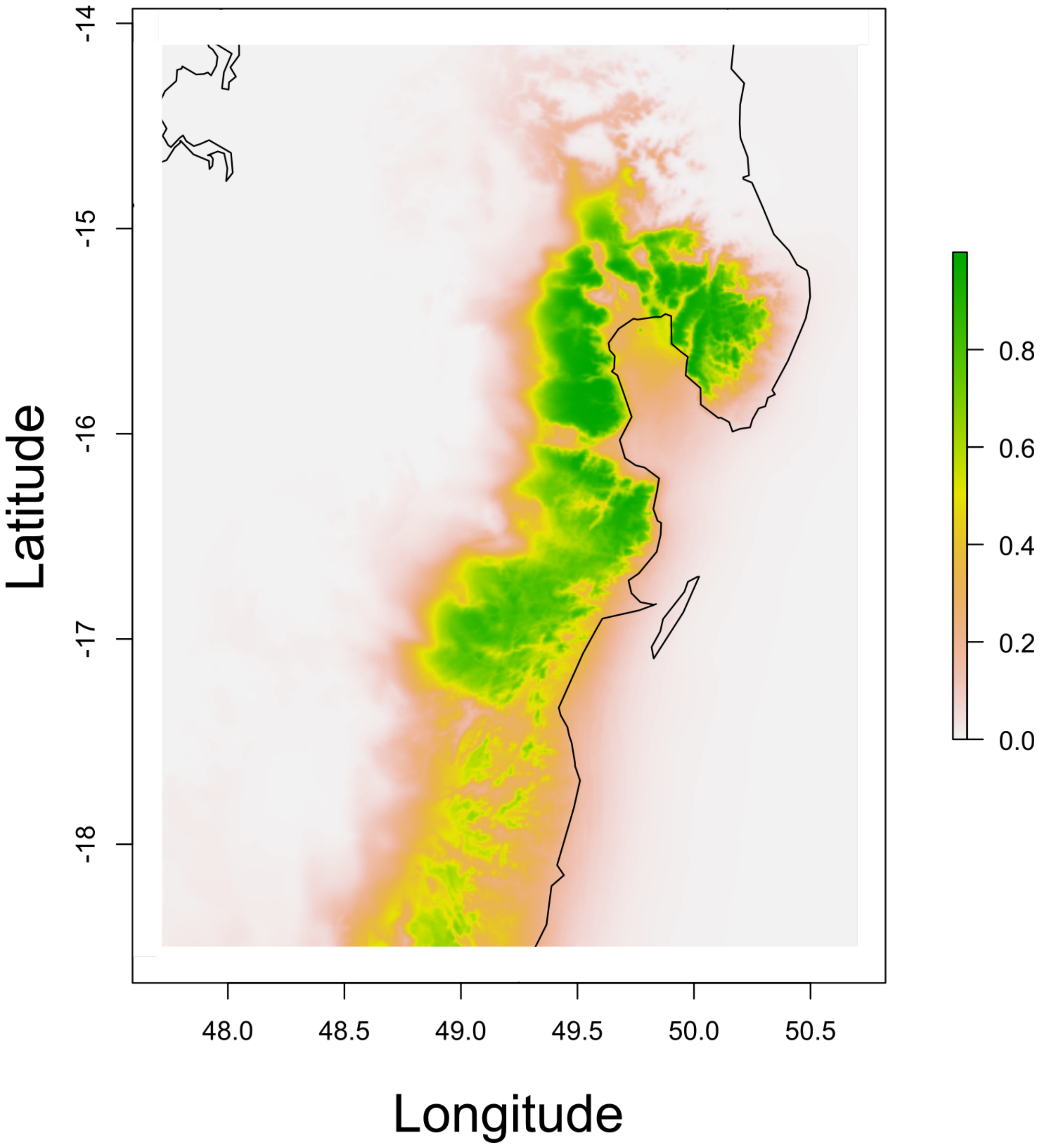


**Fig. S7:** Climatic niche suitability raster for *M. jonahi* across the entire study region (not scaled) used for isolation-by-resistance analysis. Resolution is 150 m per pixel.

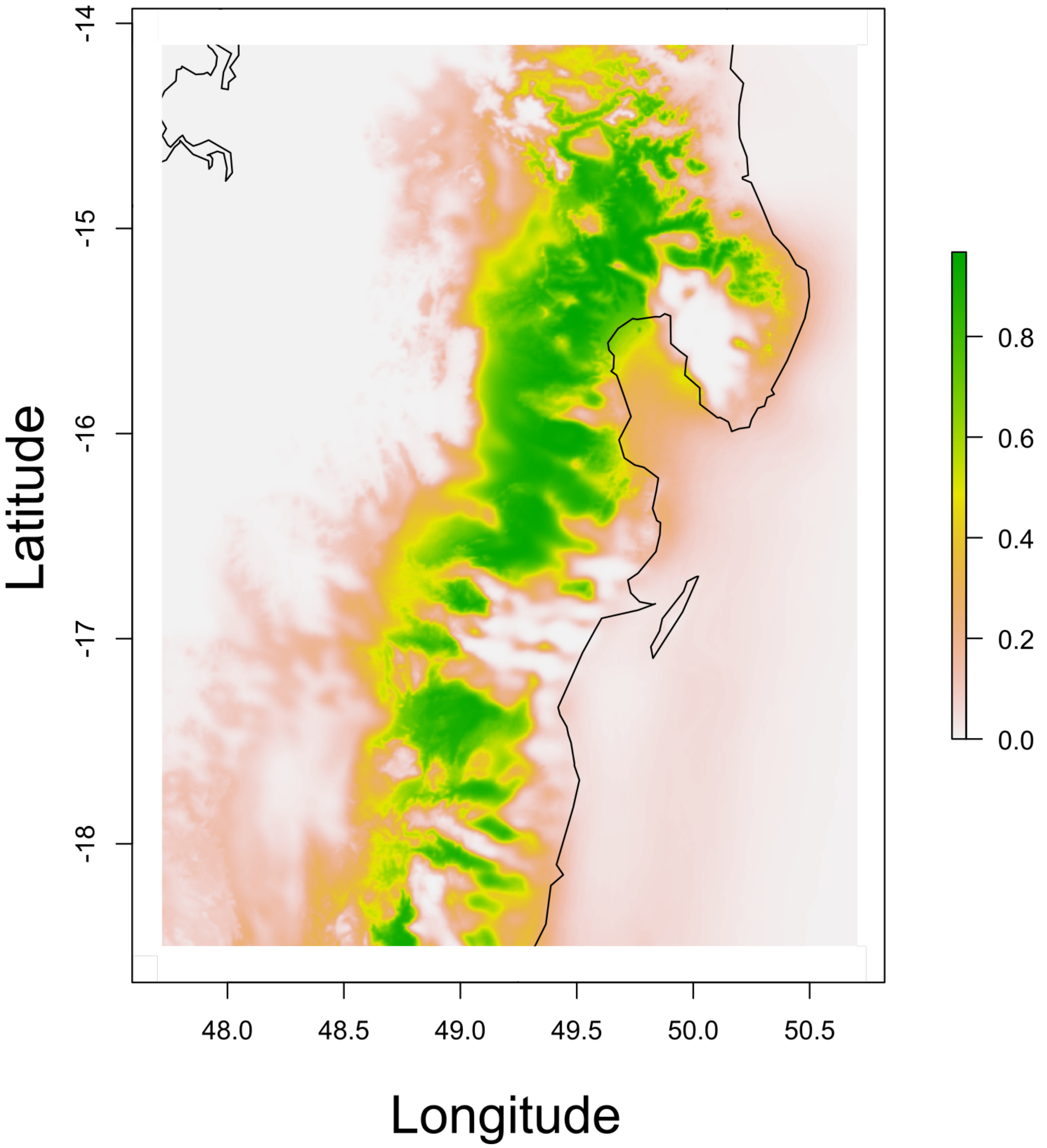


**Fig. S8:** Climatic niche suitability raster for *M. lehilahytsara* across the entire study region (not scaled) used for isolation-by-resistance analysis. Resolution is 150 m per pixel.

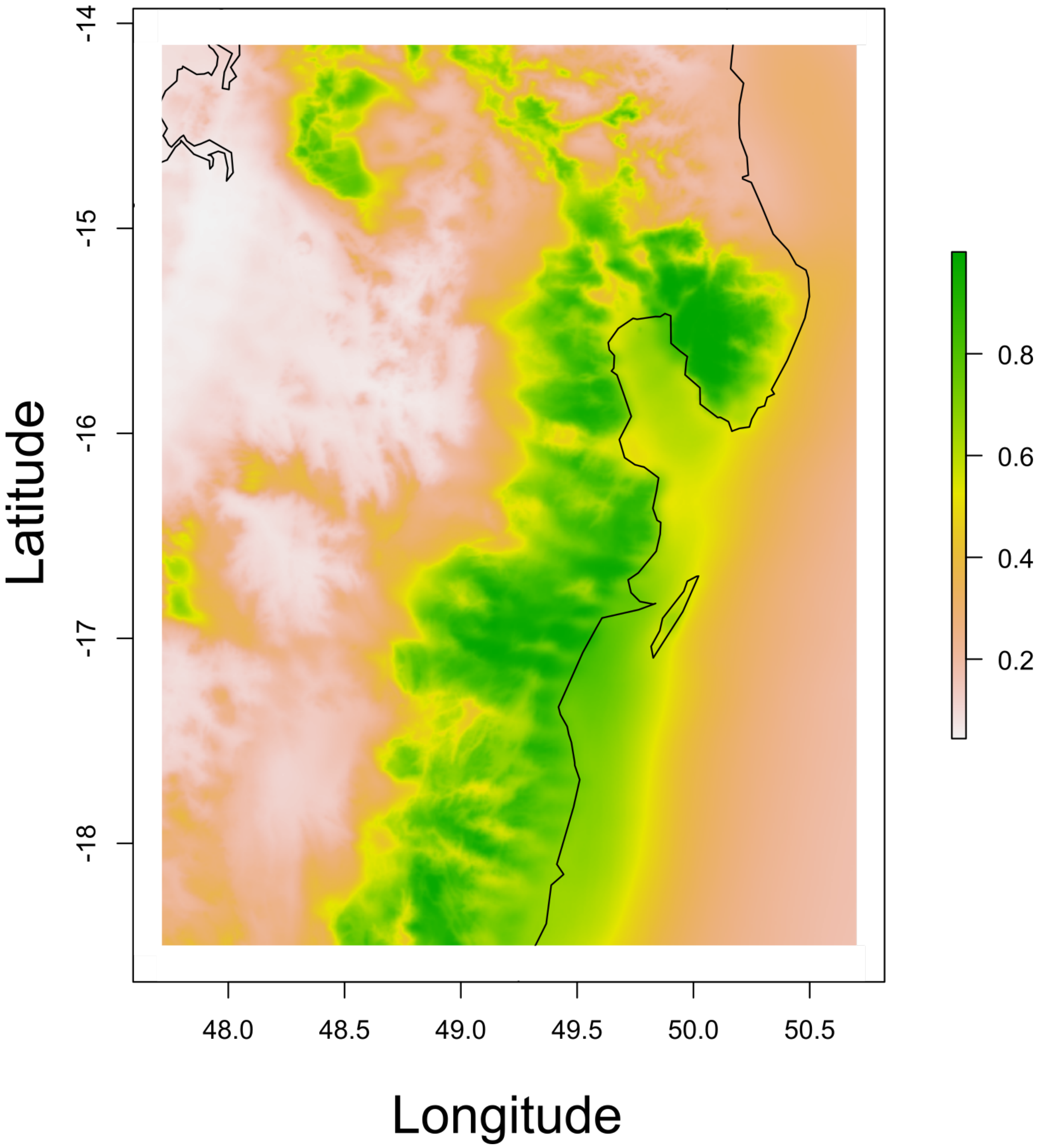


**Fig. S9:** Climatic niche suitability raster for *M. simmonsi* across the entire study region (not scaled) used for isolation-by-resistance analysis. Resolution is 150 m per pixel.

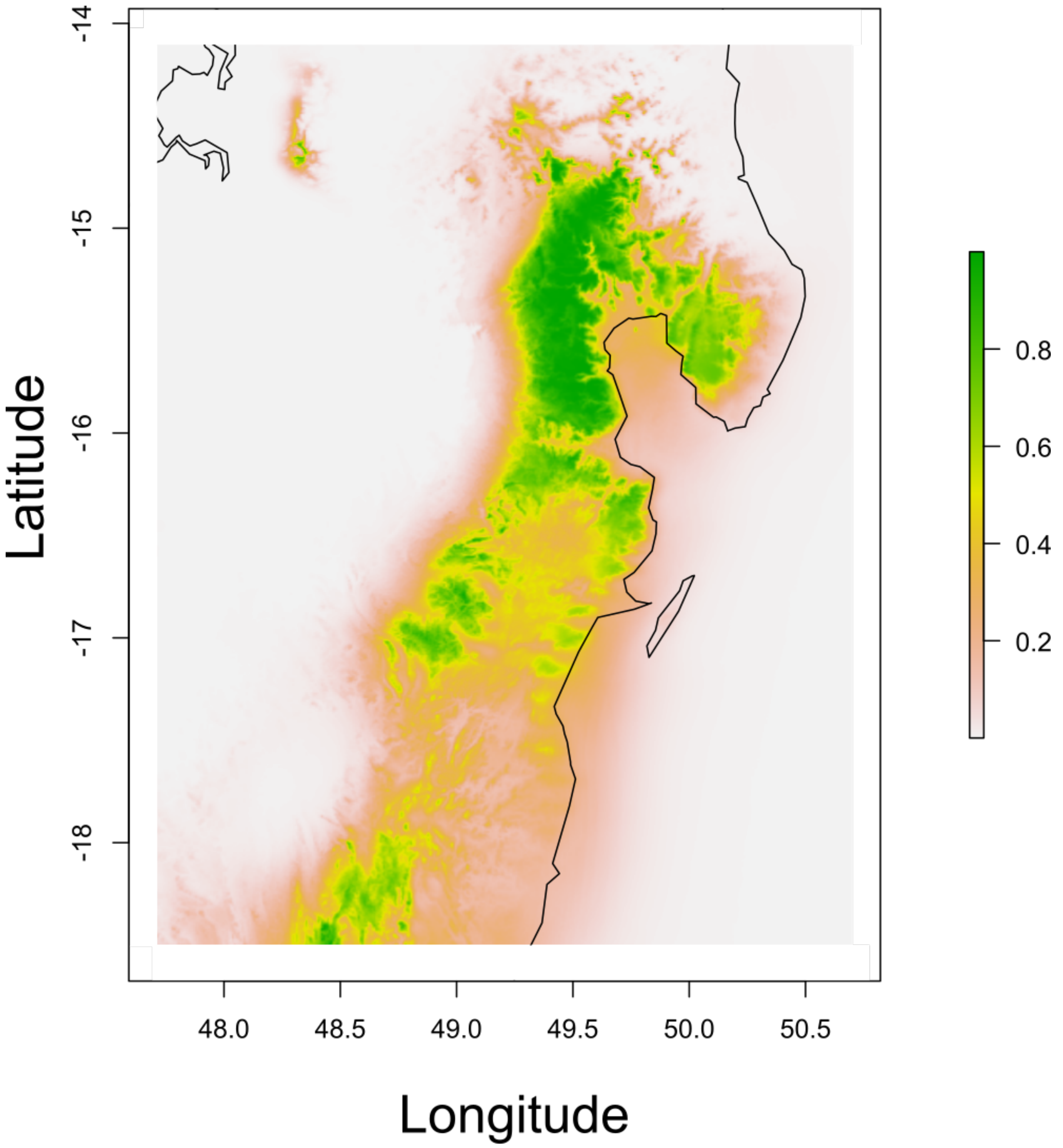


**Fig. S10:** Climatic niche suitability raster for *A. laniger* across the entire study region (not scaled) used for isolation-by-resistance analysis. Resolution is 150 m per pixel.

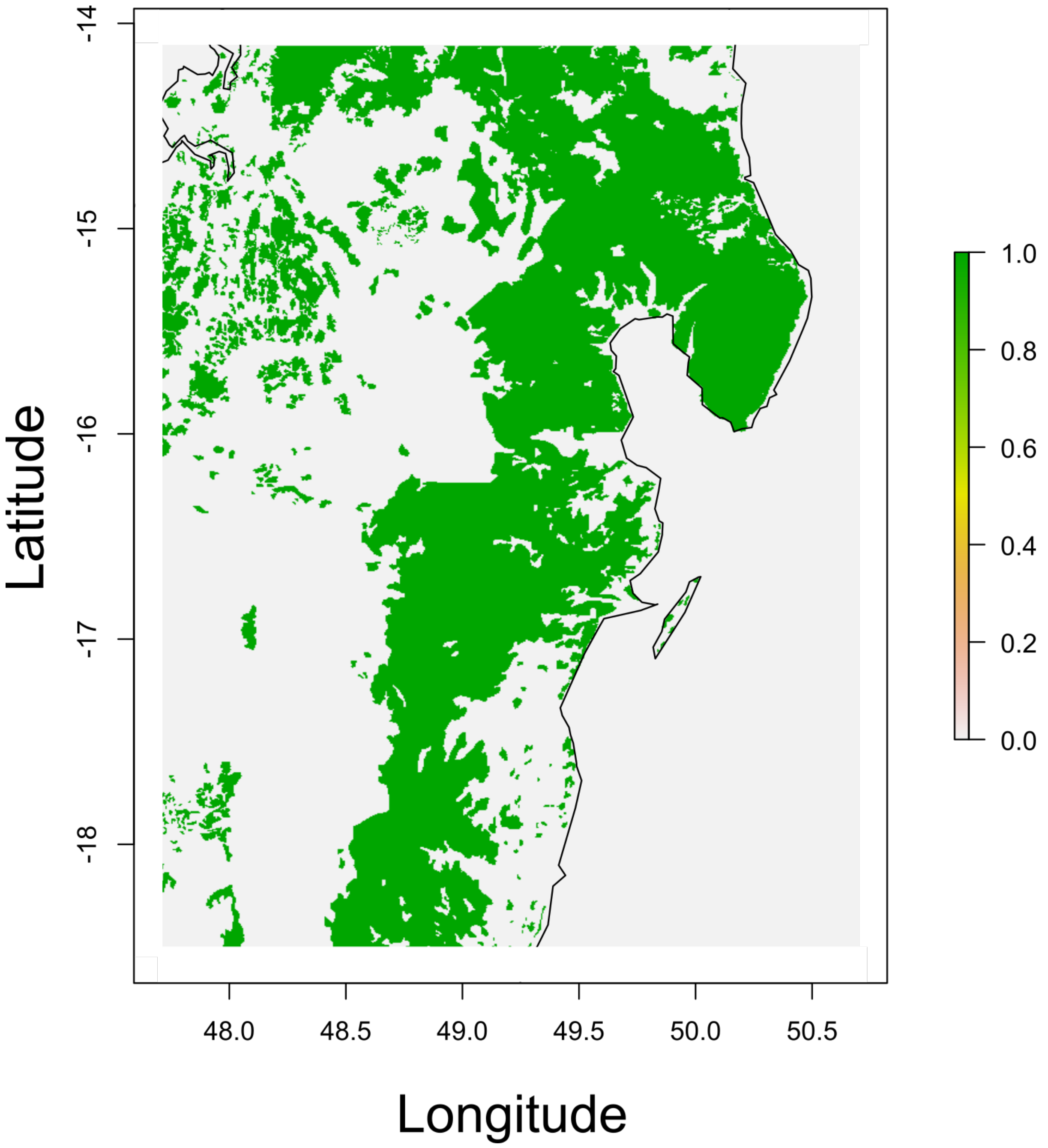


**Fig. S11:** Raster corresponding to forest cover in 1953 (Vieilledent et al., 2018) across the entire study region used for isolation-by-resistance analysis. Resolution is 150 m per pixel.

### *M. jonahi*

| | Elevation | Landscape heterogeneity | Rivers | Climatic niche suitability | Forest |
|---|---|---|---|---|---|
| Elevation | 1.00 | 0.30 | -0.40 | -0.40 | 0.57 |
| Landscape heterogeneity | 0.30 | 1.00 | -0.23 | 0.28 | -0.07 |
| Rivers | -0.40 | -0.23 | 1.00 | -0.13 | -0.01 |
| Climatic niche suitability | -0.40 | 0.28 | -0.13 | 1.00 | 0.57 |
| Forest | 0.57 | -0.07 | -0.01 | 0.57 | 1.00 |

### *M. lehilahytsara*

| | Elevation | Landscape heterogeneity | Rivers | Climatic niche suitability | Forest |
|---|---|---|---|---|---|
| Elevation | 1.00 | 0.32 | -0.27 | -0.04 | 0.13 |
| Landscape heterogeneity | 0.32 | 1.00 | -0.12 | -0.16 | 0.26 |
| Rivers | -0.27 | -0.12 | 1.00 | -0.02 | -0.10 |
| Climatic niche suitability | -0.04 | -0.16 | -0.02 | 1.00 | 0.47 |
| Forest | 0.13 | 0.26 | -0.10 | 0.47 | 1.00 |

### *M. simmonsi*

| | Elevation | Landscape heterogeneity | Rivers | Climatic niche suitability | Forest |
|---|---|---|---|---|---|
| Elevation | 1.00 | -0.13 | -0.66 | -0.36 | 0.47 |
| Landscape heterogeneity | -0.13 | 1.00 | -0.17 | -0.11 | 0.22 |
| Rivers | -0.66 | -0.17 | 1.00 | -0.27 | 0.03 |
| Climatic niche suitability | -0.36 | -0.11 | -0.27 | 1.00 | 0.39 |
| Forest | 0.47 | 0.22 | 0.03 | 0.39 | 1.00 |

### *A. laniger*

| | Elevation | Landscape heterogeneity | Rivers | Climatic niche suitability | Forest |
|---|---|---|---|---|---|
| Elevation | 1.00 | 0.18 | -0.37 | -0.14 | 0.34 |
| Landscape heterogeneity | 0.18 | 1.00 | -0.22 | 0.31 | 0.09 |
| Rivers | -0.37 | -0.22 | 1.00 | -0.04 | -0.08 |
| Climatic niche suitability | -0.14 | 0.31 | -0.04 | 1.00 | 0.53 |
| Forest | 0.34 | 0.09 | -0.08 | 0.53 | 1.00 |

**Fig. S12:** Correlation coefficients for pairwise comparisons of cropped and scaled rasters at a resolution of 150 m for each species (based on a pixel subsample of 5%). Red and blue colors indicate negative and positive correlations, respectively. Color intensity is proportional to the strength of the correlation.

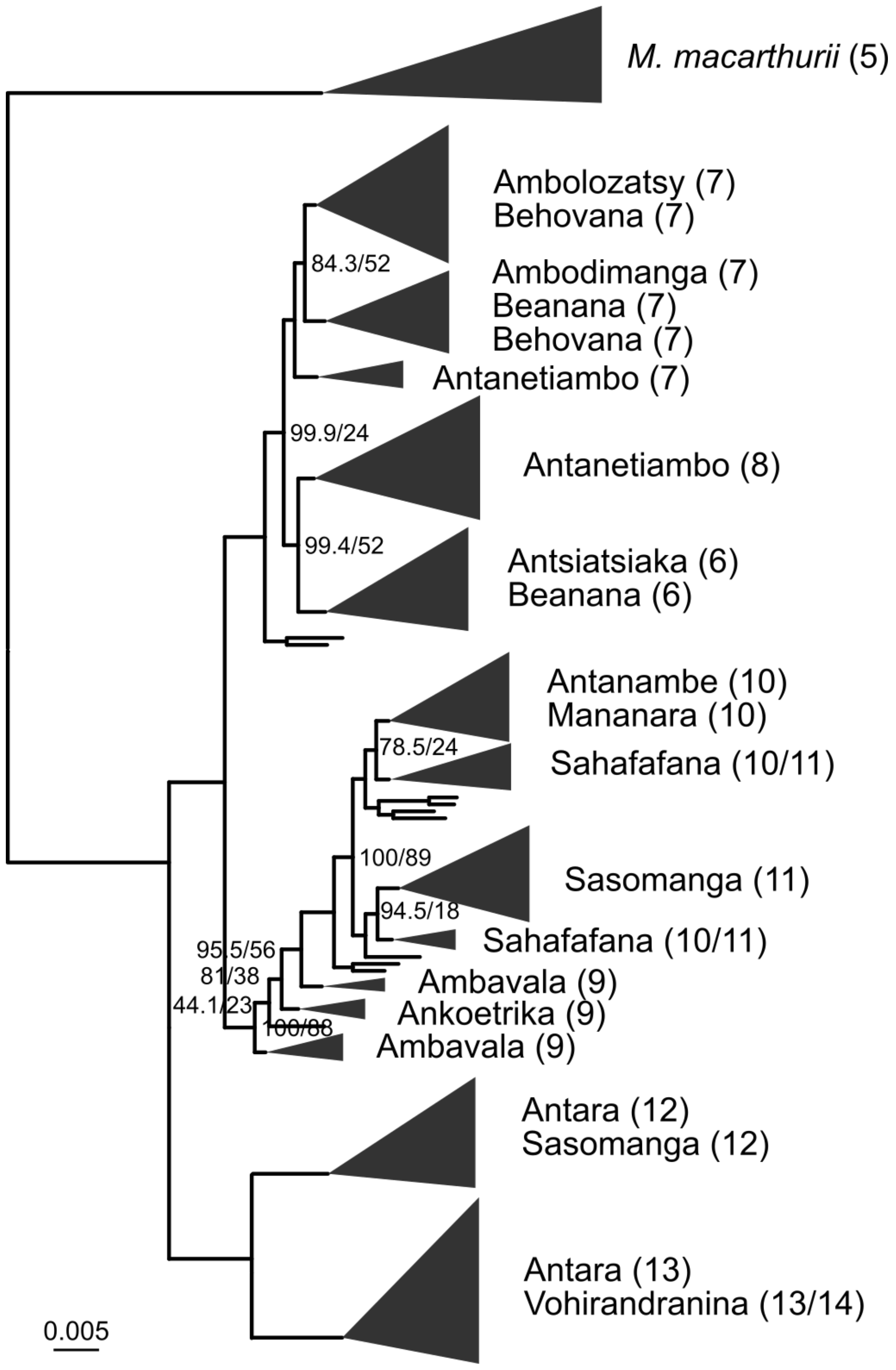


**Fig. S13:** Maximum likelihood phylogeny inferred with IQ-TREE for *M. jonahi* and *M. macarthurii*. Tip labels denote population names (of *M. jonahi* if not mentioned otherwise) and associated inter-river systems (in parentheses). Triangles represent collapsed tips proportional to sample size. Node labels represent ultrafast bootstrap and SH-like approximate likelihood ratio (SH-aLRT) support, given only for major clades and if below 100. Scale is substitutions per site.

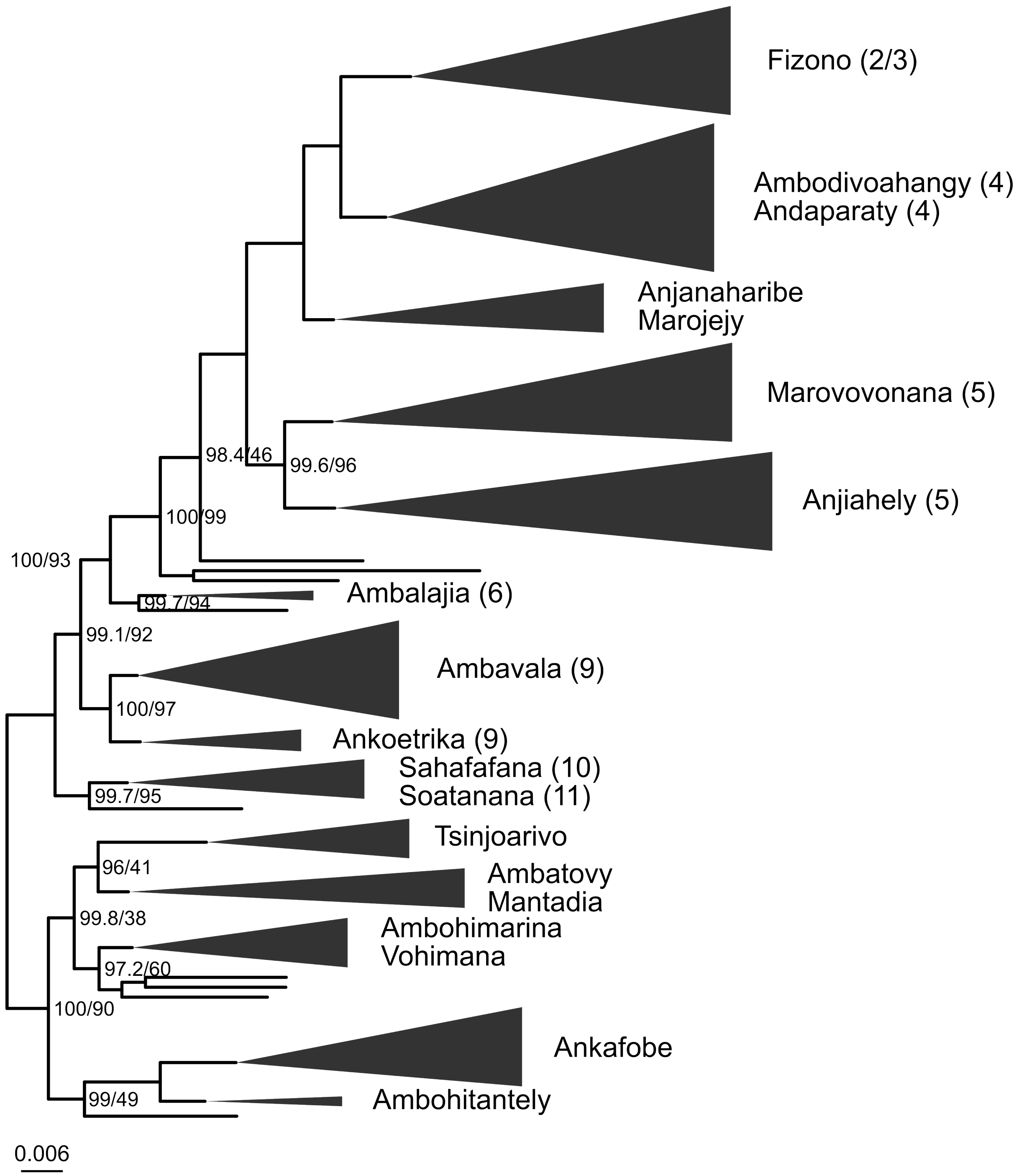


**Fig. S14:** Maximum likelihood phylogeny inferred with IQ-TREE for *M. lehilahytsara*. Tip labels denote population names and associated inter-river systems if applicable (in parentheses). Triangles represent collapsed tips proportional to sample size. Node labels represent ultrafast bootstrap and SH-like approximate likelihood ratio (SH-aLRT) support, given only for major clades and if below 100. Scale is substitutions per site.

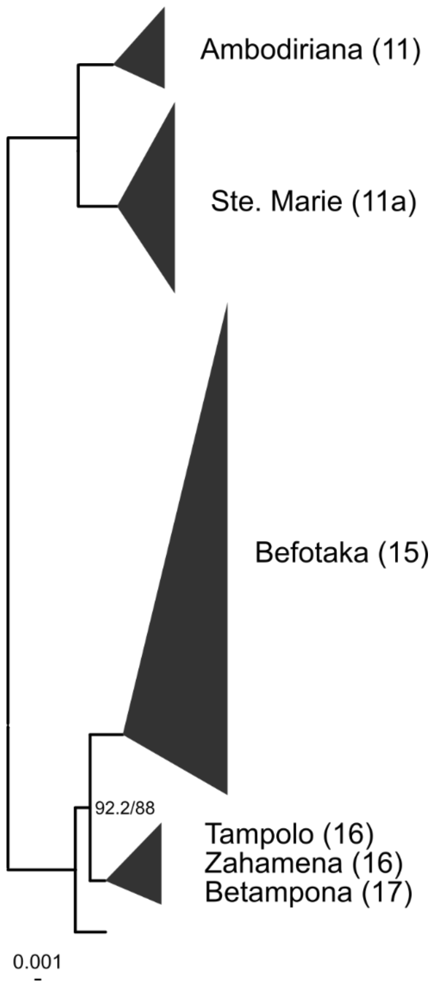


**Fig. S15:** Maximum likelihood phylogeny inferred with IQ-TREE for *M. simmonsi*. Tip labels denote population names and associated inter-river systems (in parentheses). Triangles represent collapsed tips proportional to sample size. Node labels represent ultrafast bootstrap and SH-like approximate likelihood ratio (SH-aLRT) support, given only for major clades and if below 100. Scale is substitutions per site.

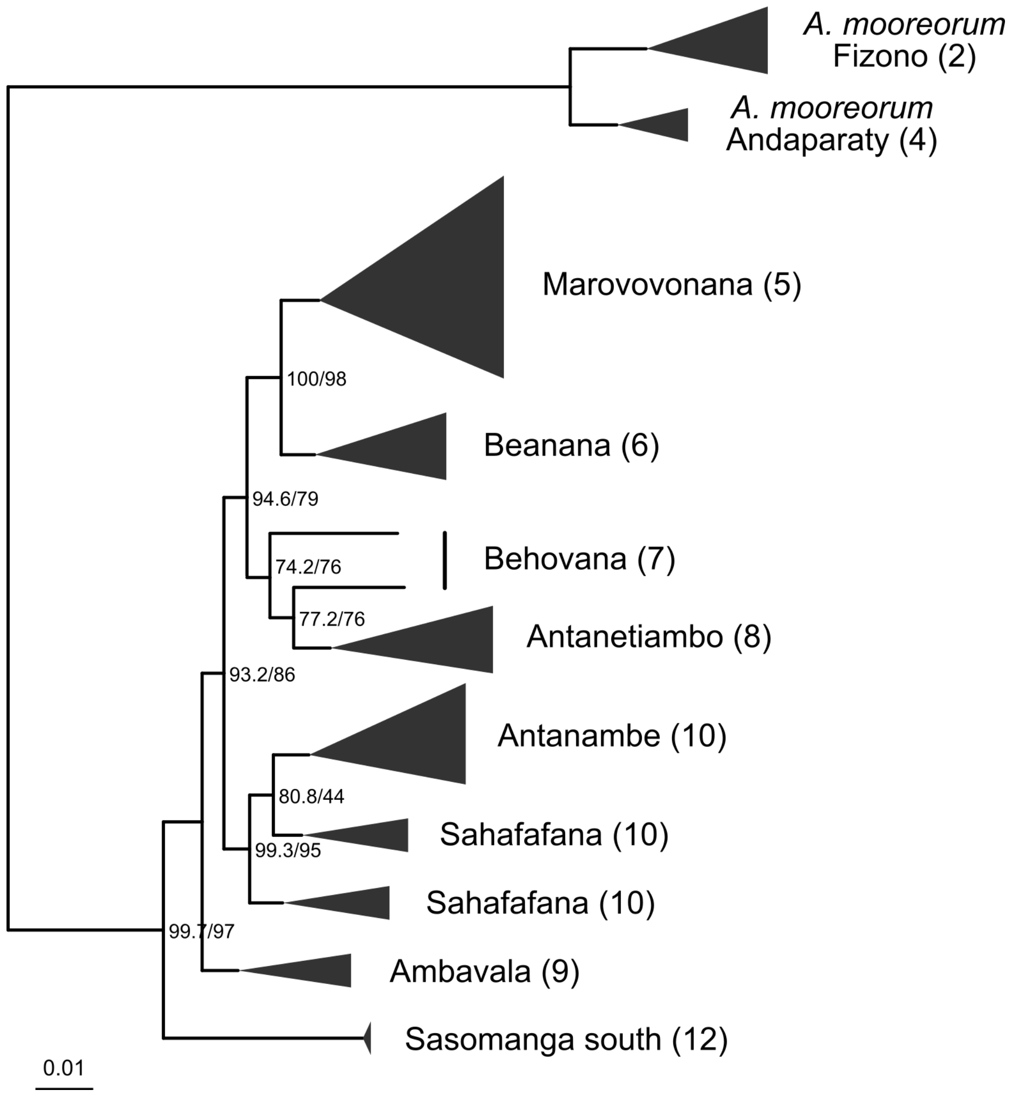


**Fig. S16:** Maximum likelihood phylogeny inferred with IQ-TREE for *A. laniger* and *A. mooreorum*. Tip labels denote population names (of *A. laniger* if not mentioned otherwise) and associated inter-river systems (in parentheses). Triangles represent collapsed tips proportional to sample size. Node labels represent ultrafast bootstrap and SH-like approximate likelihood ratio (SH-aLRT) support, given only for major clades and if below 100. Scale is substitutions per site.

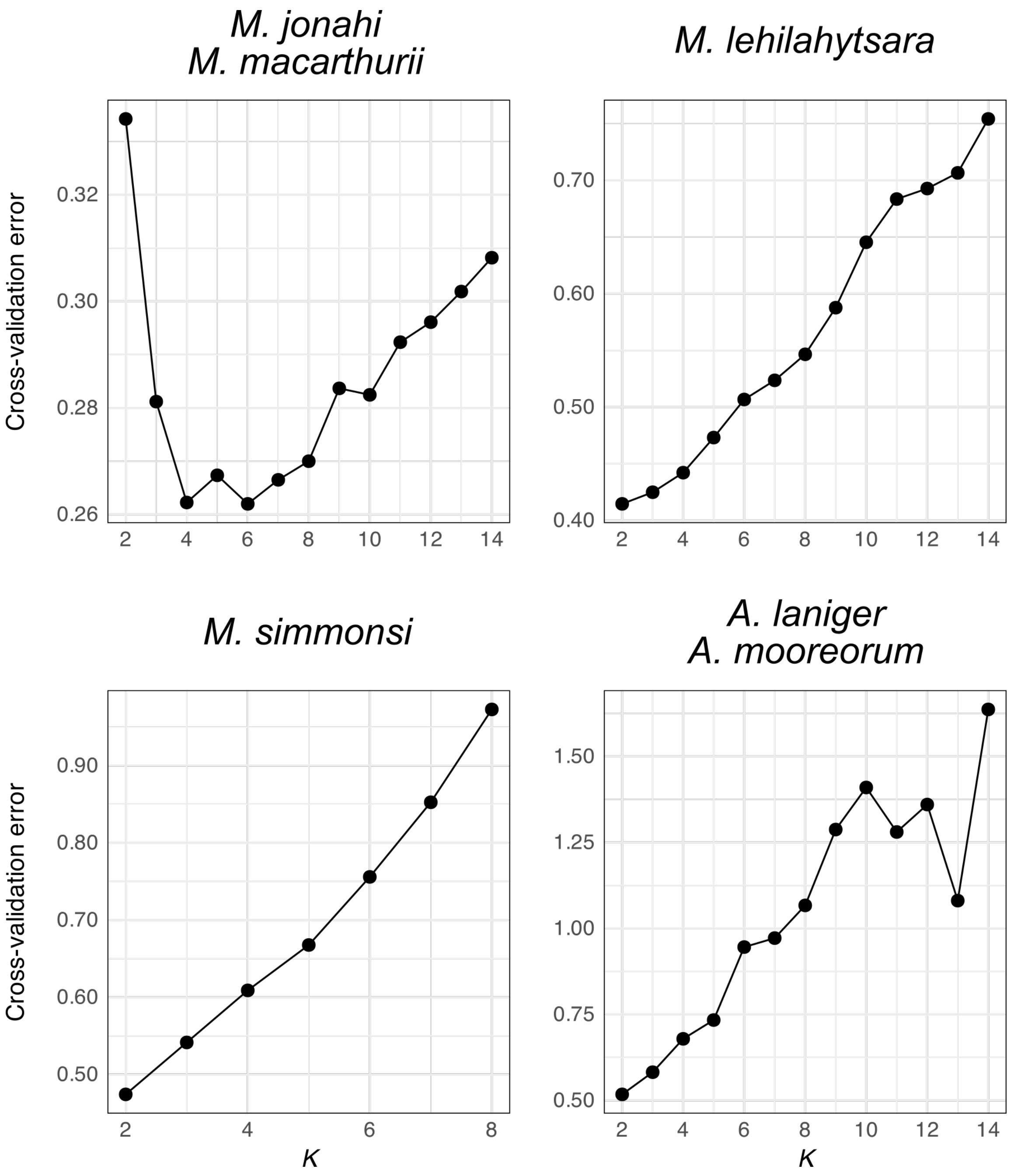


**Fig. S17:** Cross-validation errors for different numbers of clusters (*K*) in clustering analysis with ADMIXTURE.

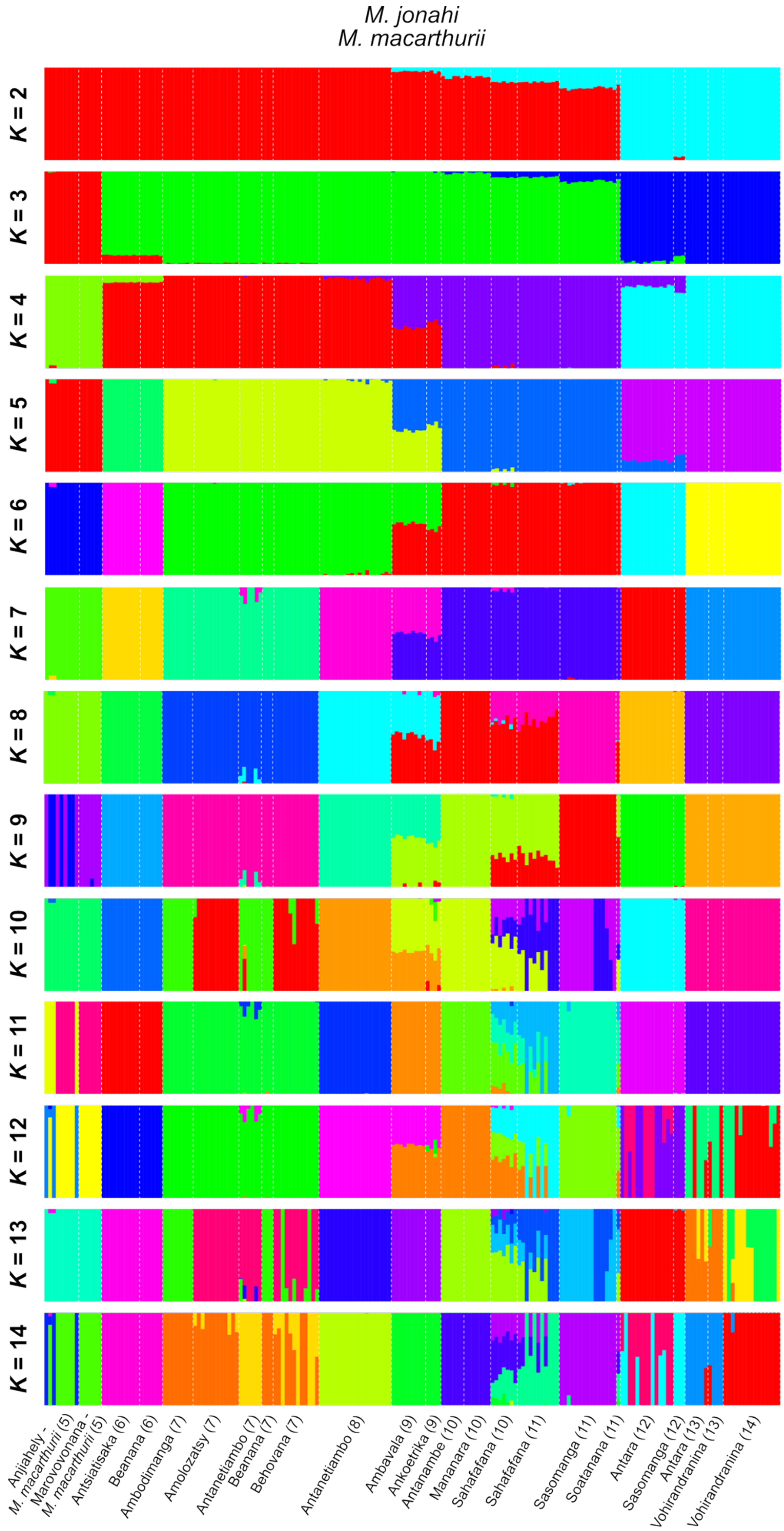


**Fig. S18:** Clustering proportions of *M. jonahi* and *M. macarthurii* individuals (columns) estimated with ADMIXTURE for two to 14 clusters (*K*). Population names refer to *M. jonahi* if not mentioned otherwise. Numbers after population names denote inter-river systems.

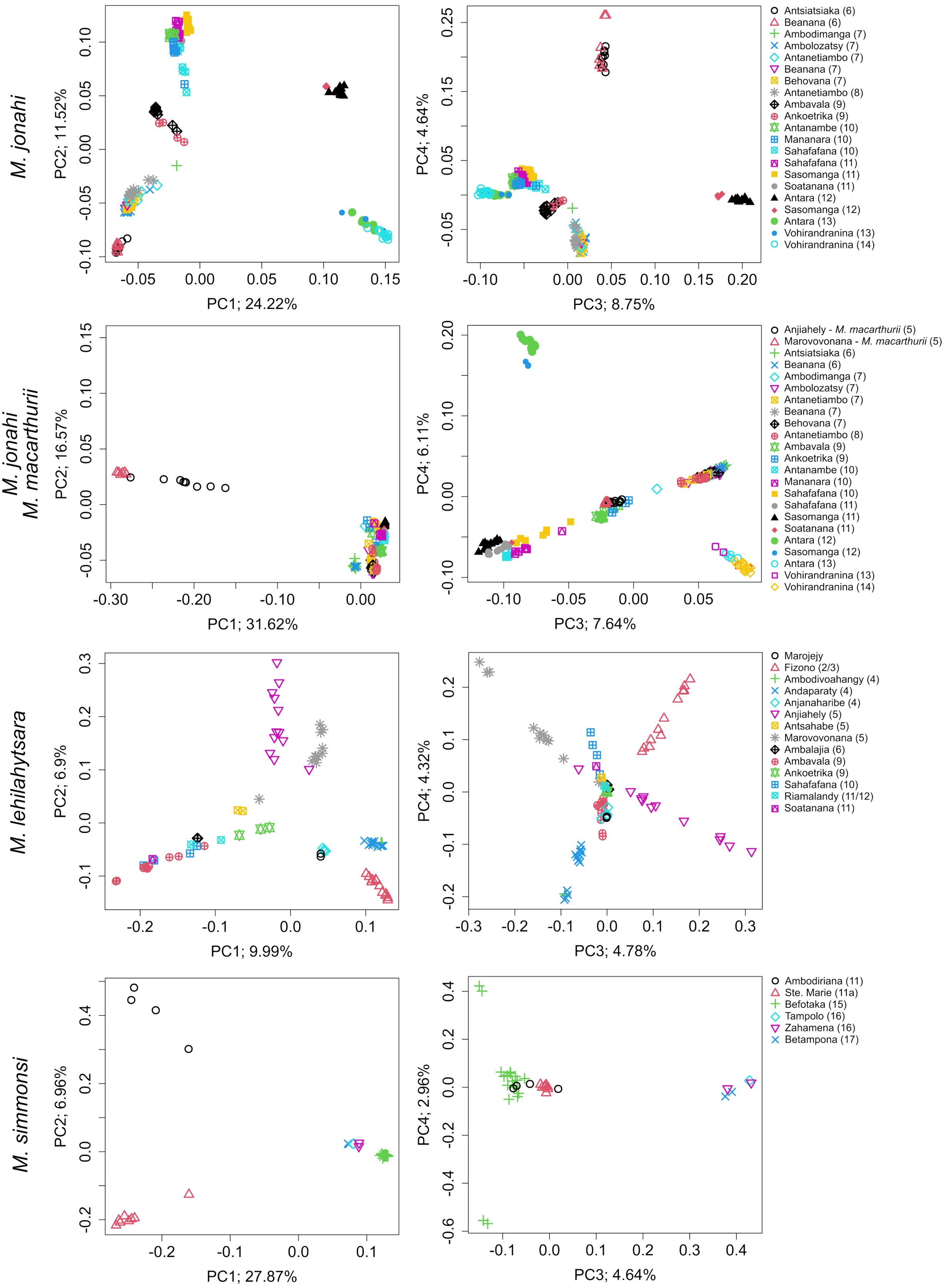


**Fig. S19:** Principal component analyses (PCA) of genetic data of *Microcebus* individuals. Left column: PC1 plotted against PC2; Right column: PC3 plotted against PC4. Percentages indicate the amount of variation explained by the PC. In plots including *M. jonahi*, population names refer to *M. jonahi* if not mentioned otherwise. Numbers after population names denote inter-river systems.

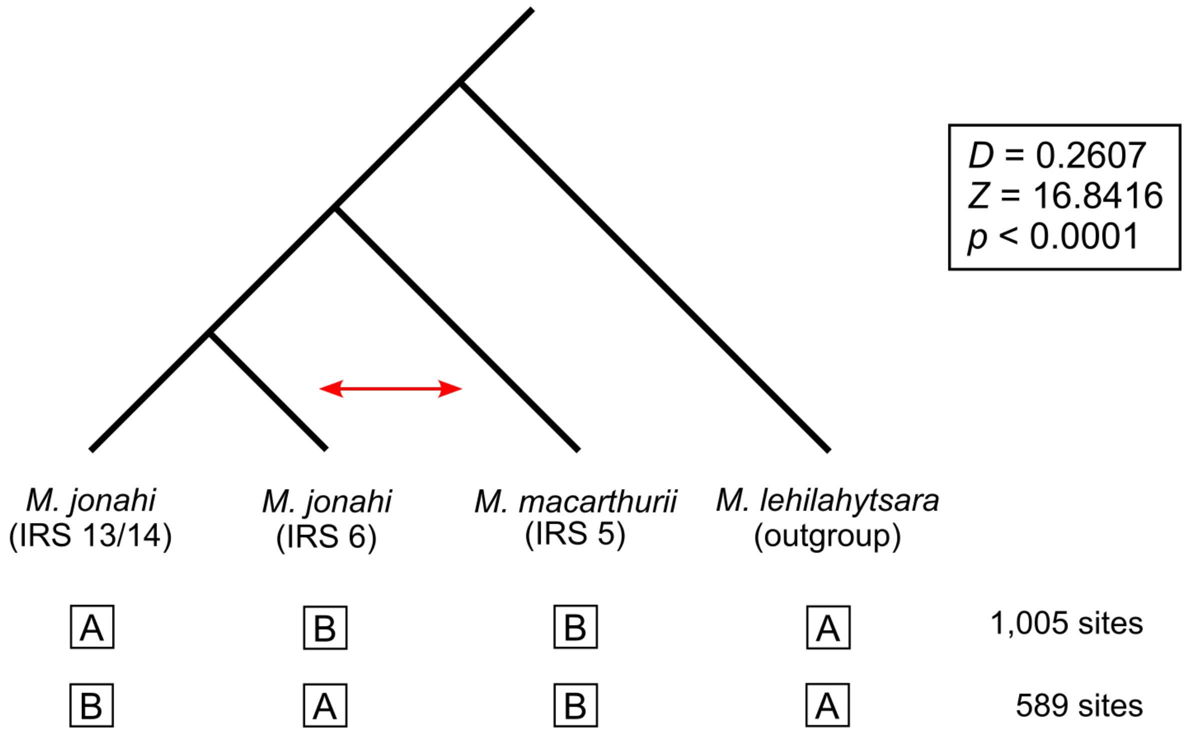


**Fig. S20:** Test for excess allele sharing between *M. macarthurii* (inter-river system/IRS 5) and the *M. jonahi* populations from IRS 6 (indicated by red arrow) via Patterson's *D* statistic. Significantly more shared sites were found between these clades (ABBA) than between *M. macarthurii* and the *M. jonahi* populations from IRSs 12 and 13 (BABA).

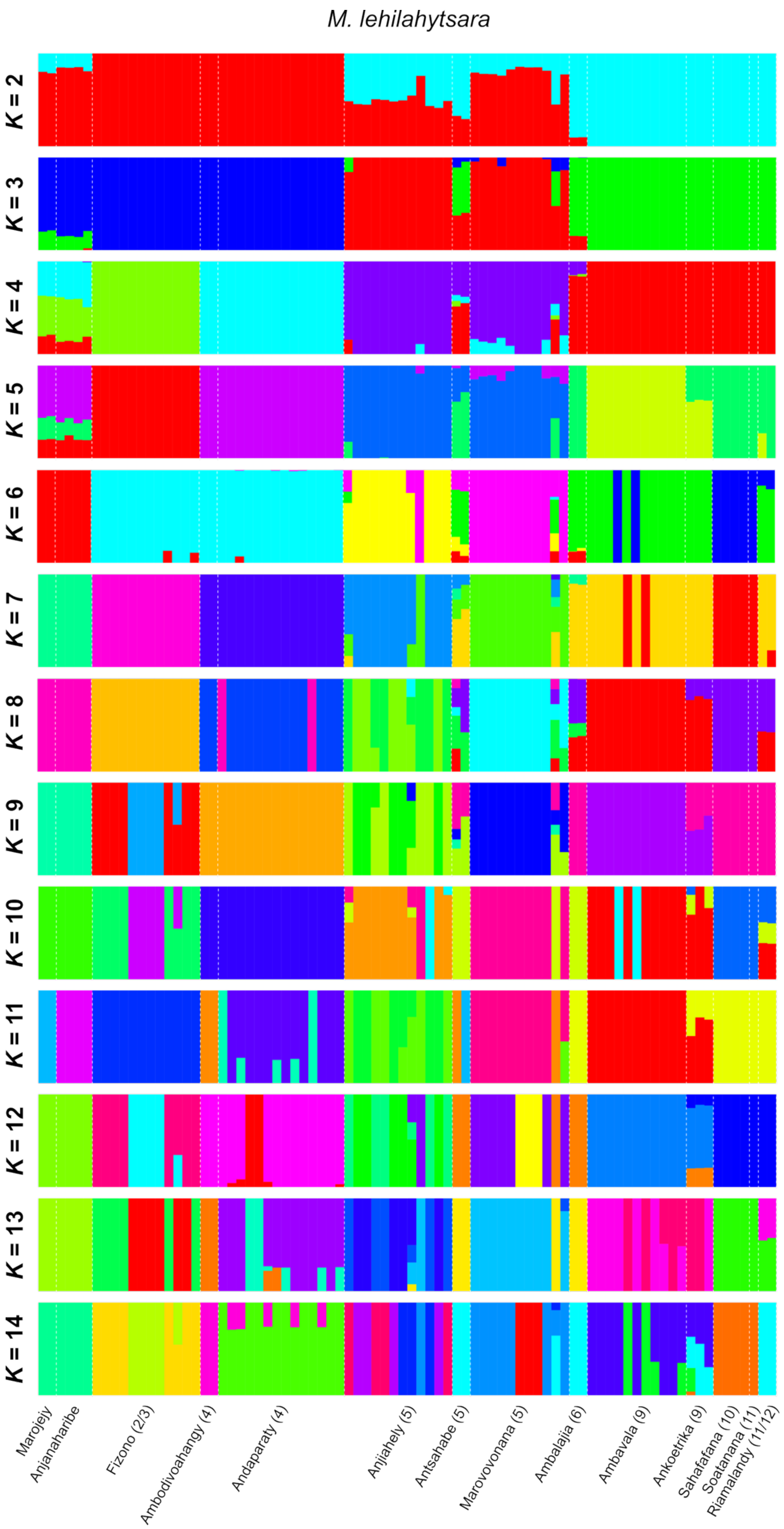


**Fig. S21:** Clustering proportions of *M. lehilahytsara* individuals (columns) estimated with ADMIXTURE for two to 14 clusters (*K*). Numbers after population names denote inter-river systems.

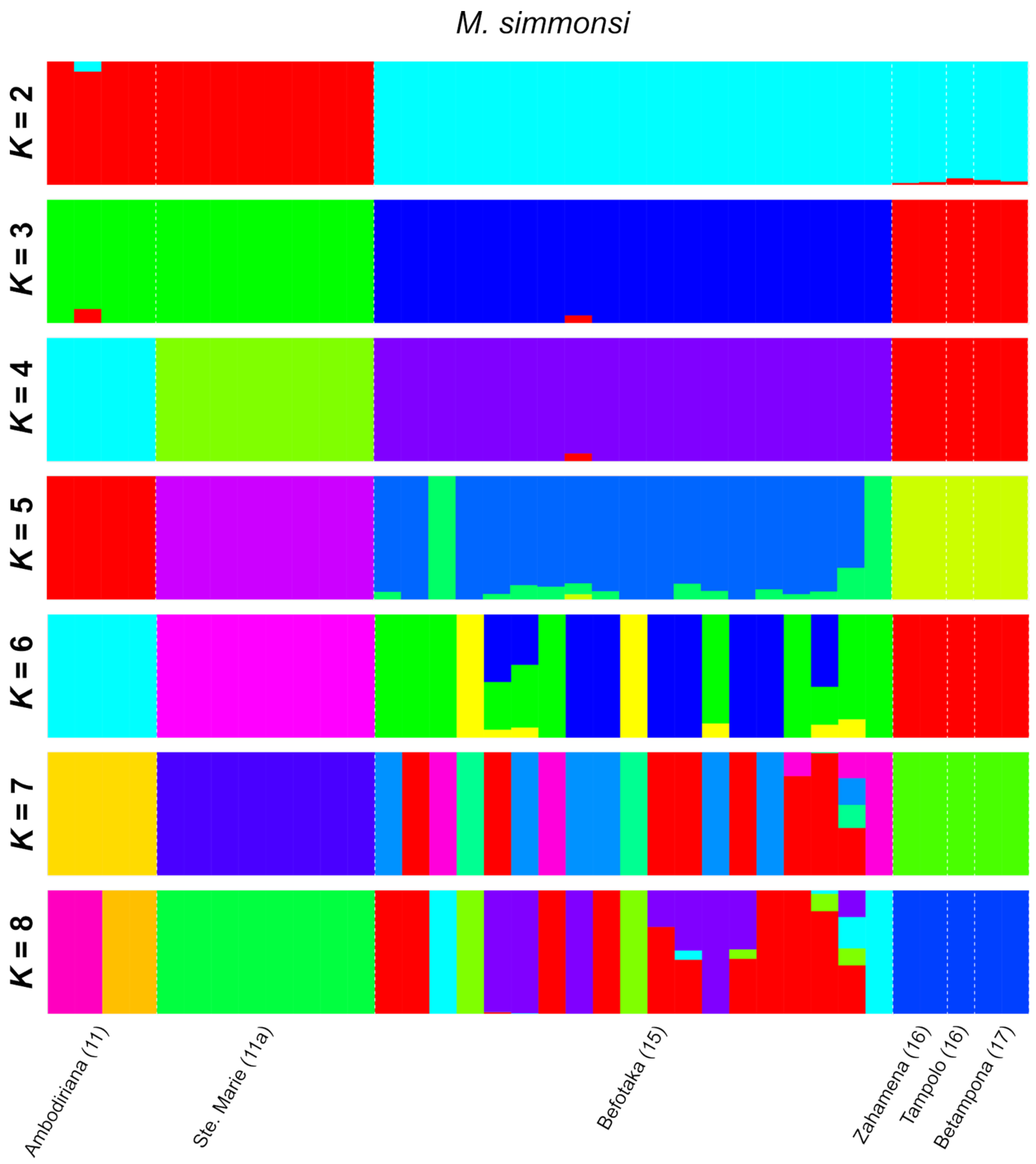


**Fig. S22:** Clustering proportions of *M. simmonsi* individuals (columns) estimated with ADMIXTURE for two to eight clusters (*K*). Numbers after population names denote inter-river systems.

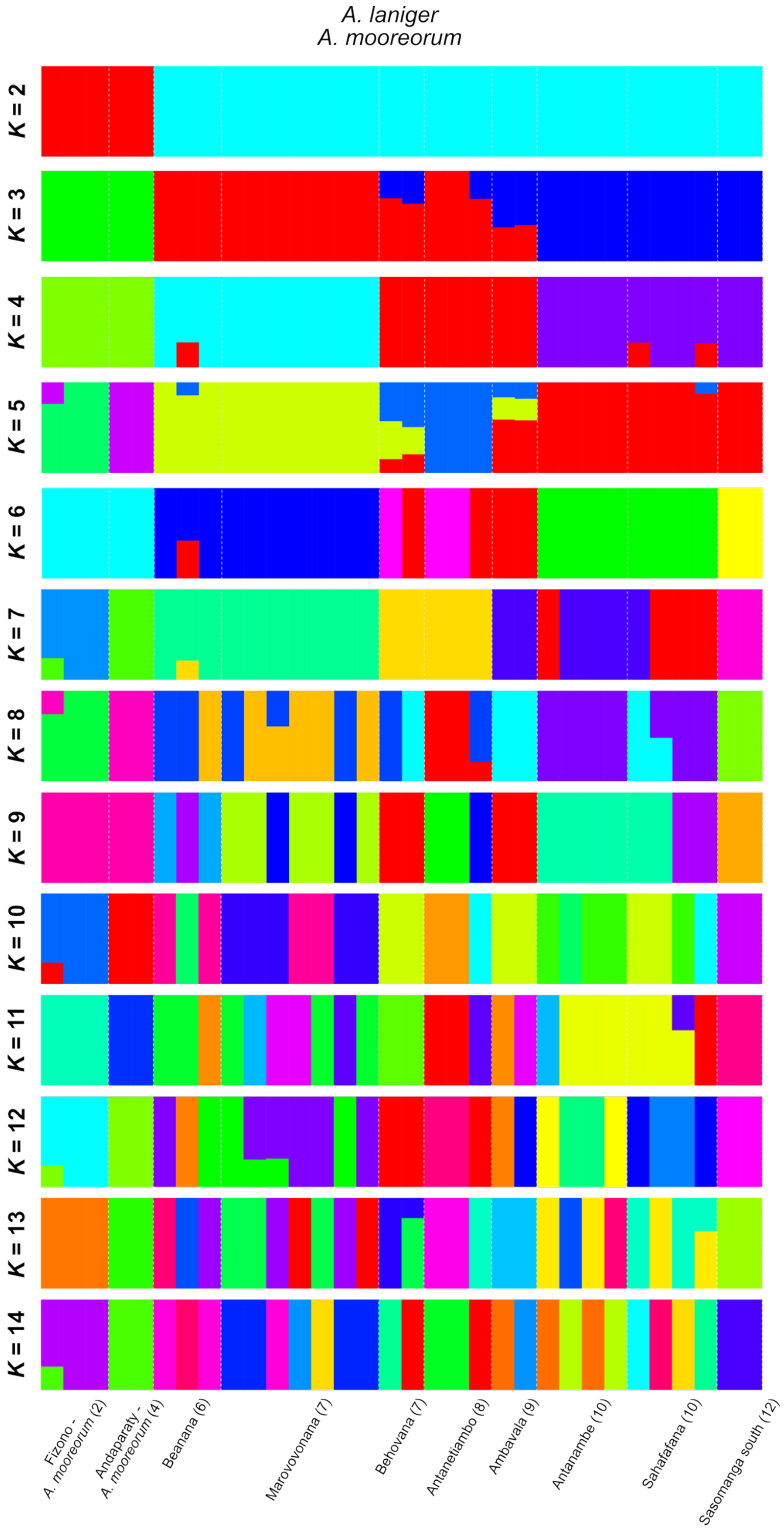


**Fig. S23:** Clustering proportions of *A. laniger* and *A. mooreorum* individuals (columns) estimated with ADMIXTURE for two to 14 clusters (*K*). Population names refer to *A.laniger* if not mentioned otherwise. Numbers after population names denote inter-river systems.

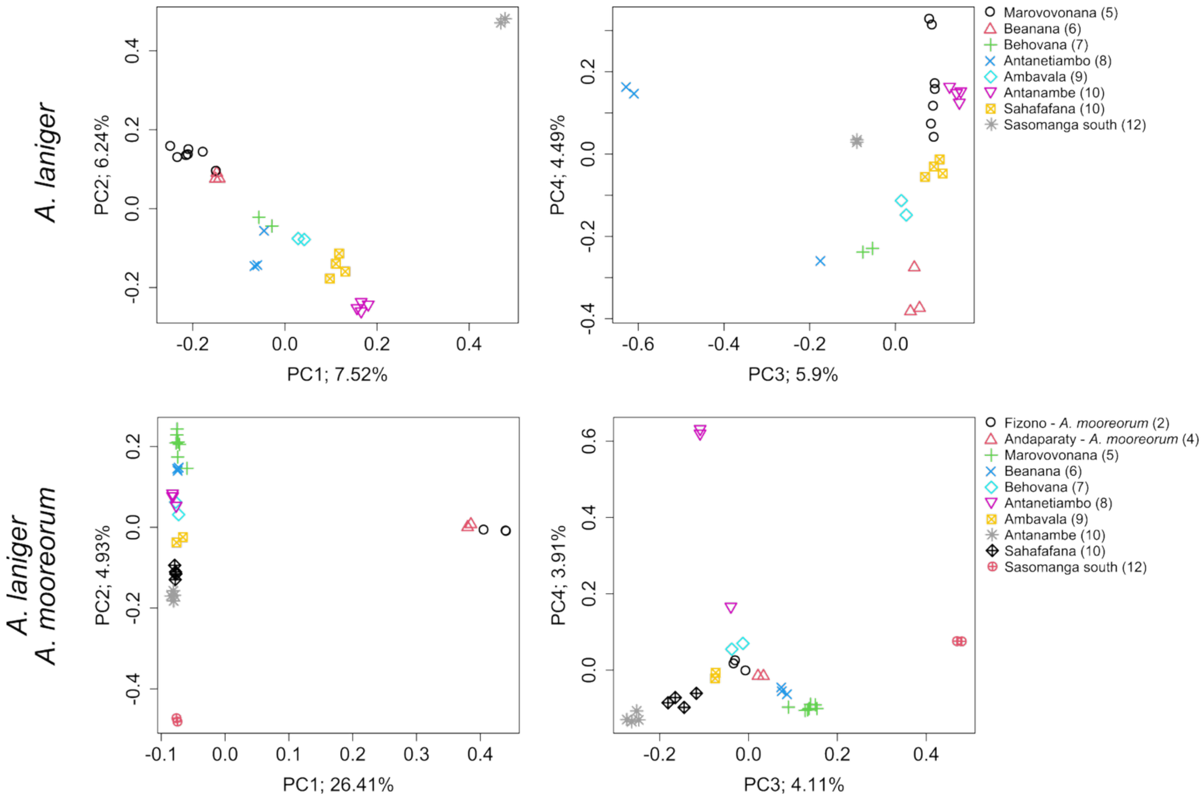


**Fig. S24:** Principal component analyses (PCA) of genetic data of *Avahi* individuals. Left column: PC1 plotted against PC2; Right column: PC3 plotted against PC4. Percentages indicate amount of variation explained by the PC. Population names refer to *A. laniger* if not mentioned otherwise. Numbers after population names denote inter-river systems.

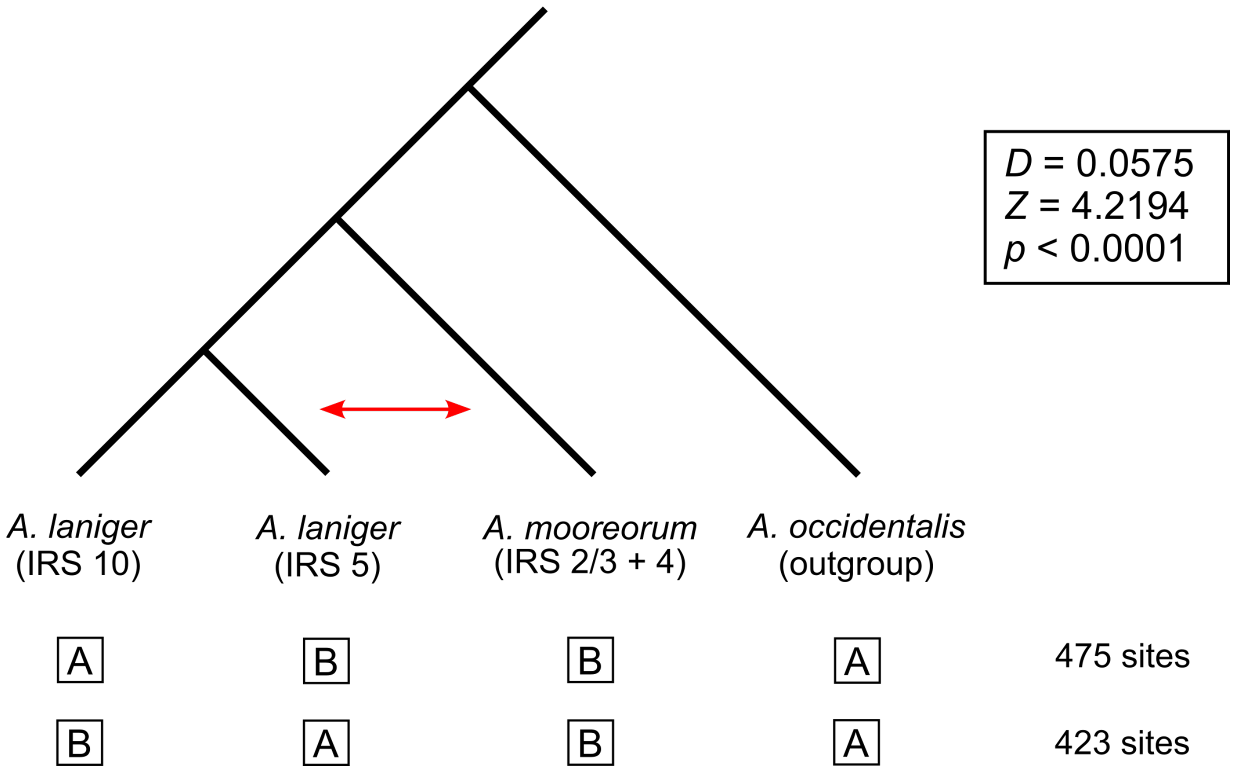


**Fig. S25:** Test for excess allele sharing between *A. laniger* (inter-river system/IRS 5) and the *A. mooreorum* populations from IRSs 2 and 4 (indicated by red arrow) via Patterson's *D* statistic. Significantly more shared sites were found between these clades (ABBA) than between *A. mooreorum* and the *A. laniger* populations from IRS 10 (BABA).

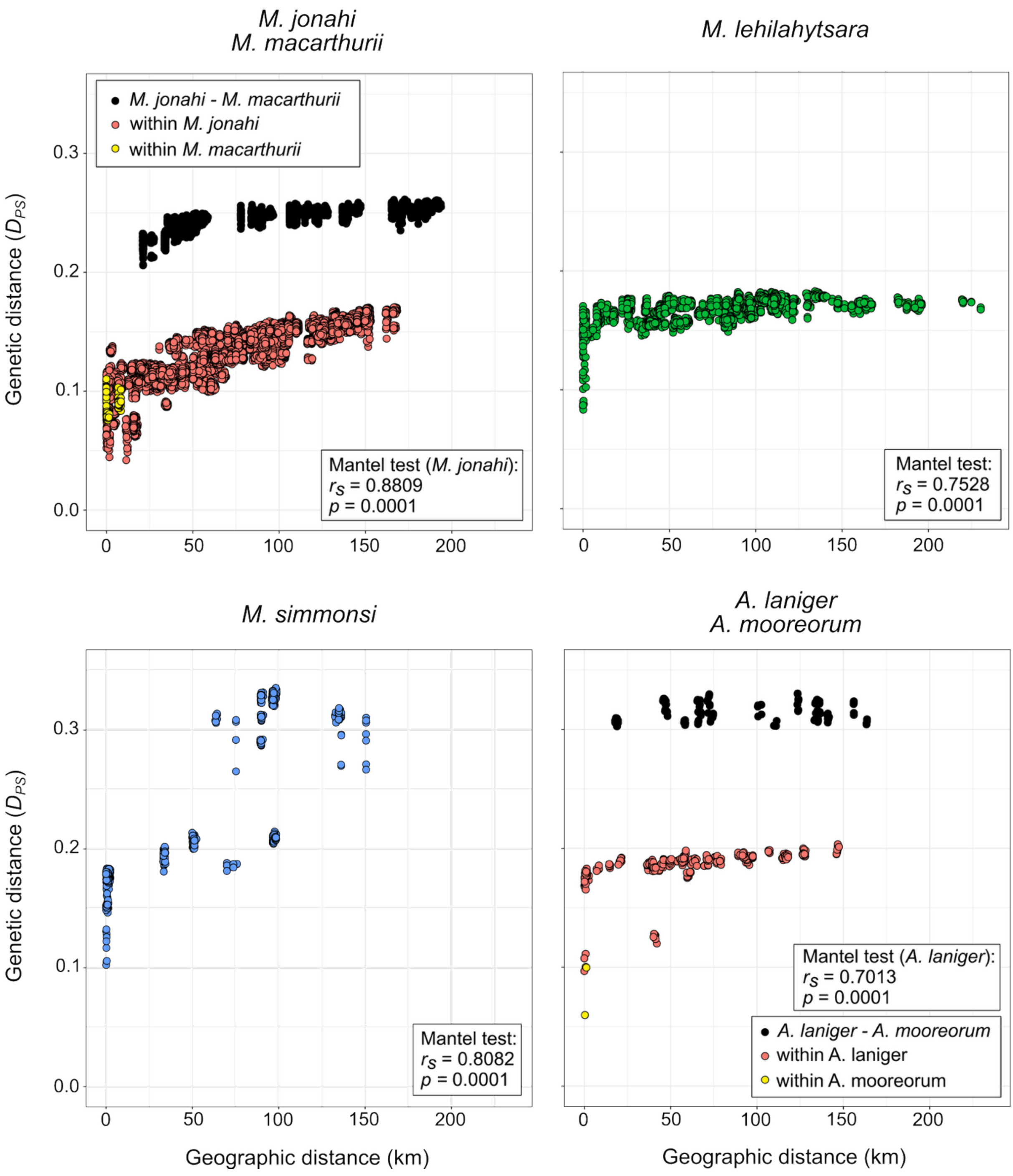


**Fig. S26:** Genetic distance $D_{PS}$ (1 – proportion of shared alleles) between individuals of the study species plotted against geographic distance. Mantel tests based on Spearman's rank correlation $r_s$ were performed to test for isolation-by-distance with 9999 permutations. The tests for *M. jonahi* and *A. laniger* were conducted without *M. macarthurii* and *A. mooreorum* populations, respectively.

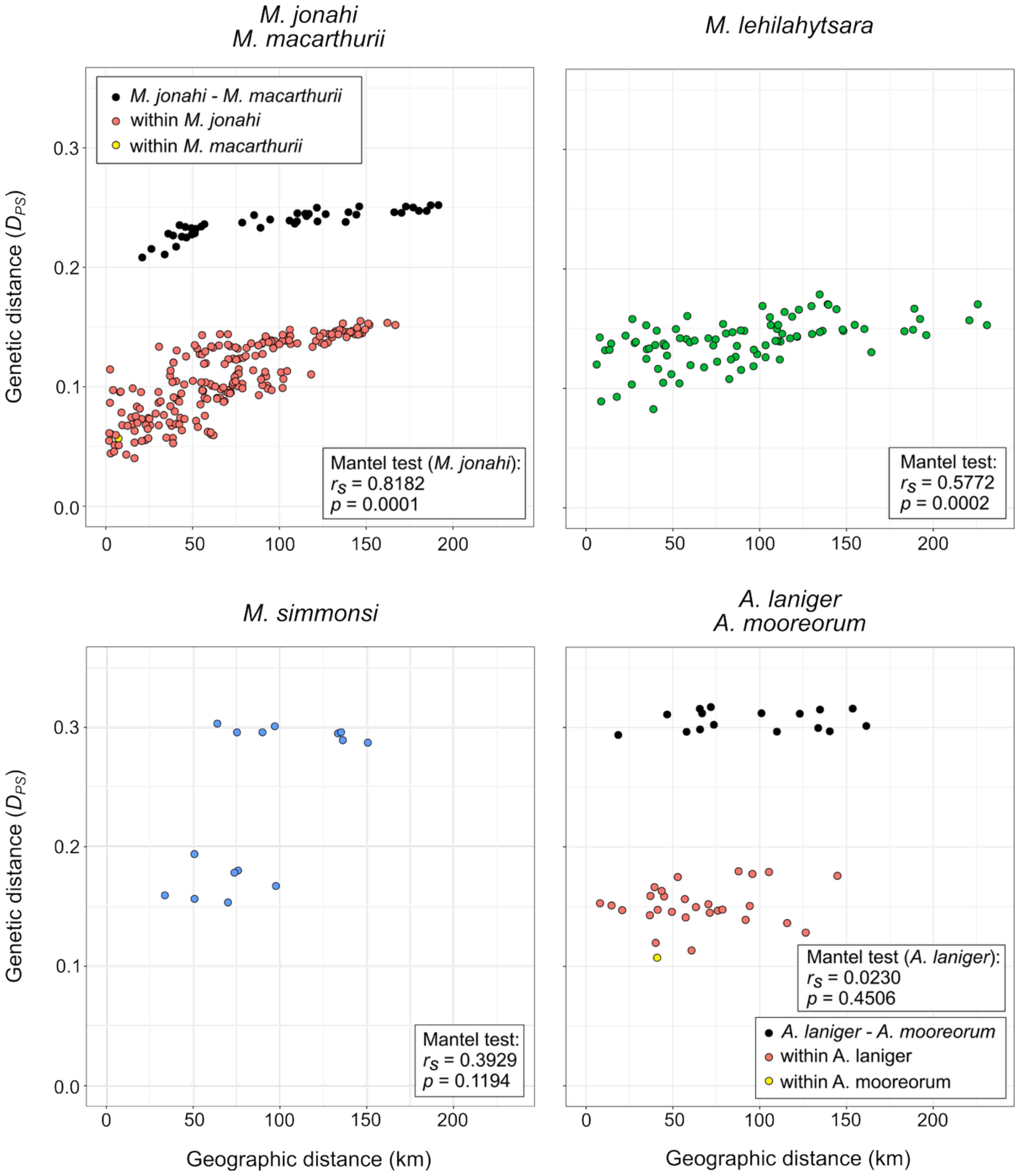


**Fig. S27:** Genetic distance $D_{PS}$ (1 – proportion of shared alleles) between populations of the study species plotted against geographic distance. Geographic distance between populations was calculated as the mean of individual distances. Mantel tests based on Spearman's rank correlation $r_s$ were performed to test for isolation-by-distance with 9999 permutations. The tests for *M. jonahi* and *A. laniger* were conducted without *M. macarthurii* and *A. mooreorum* populations, respectively.

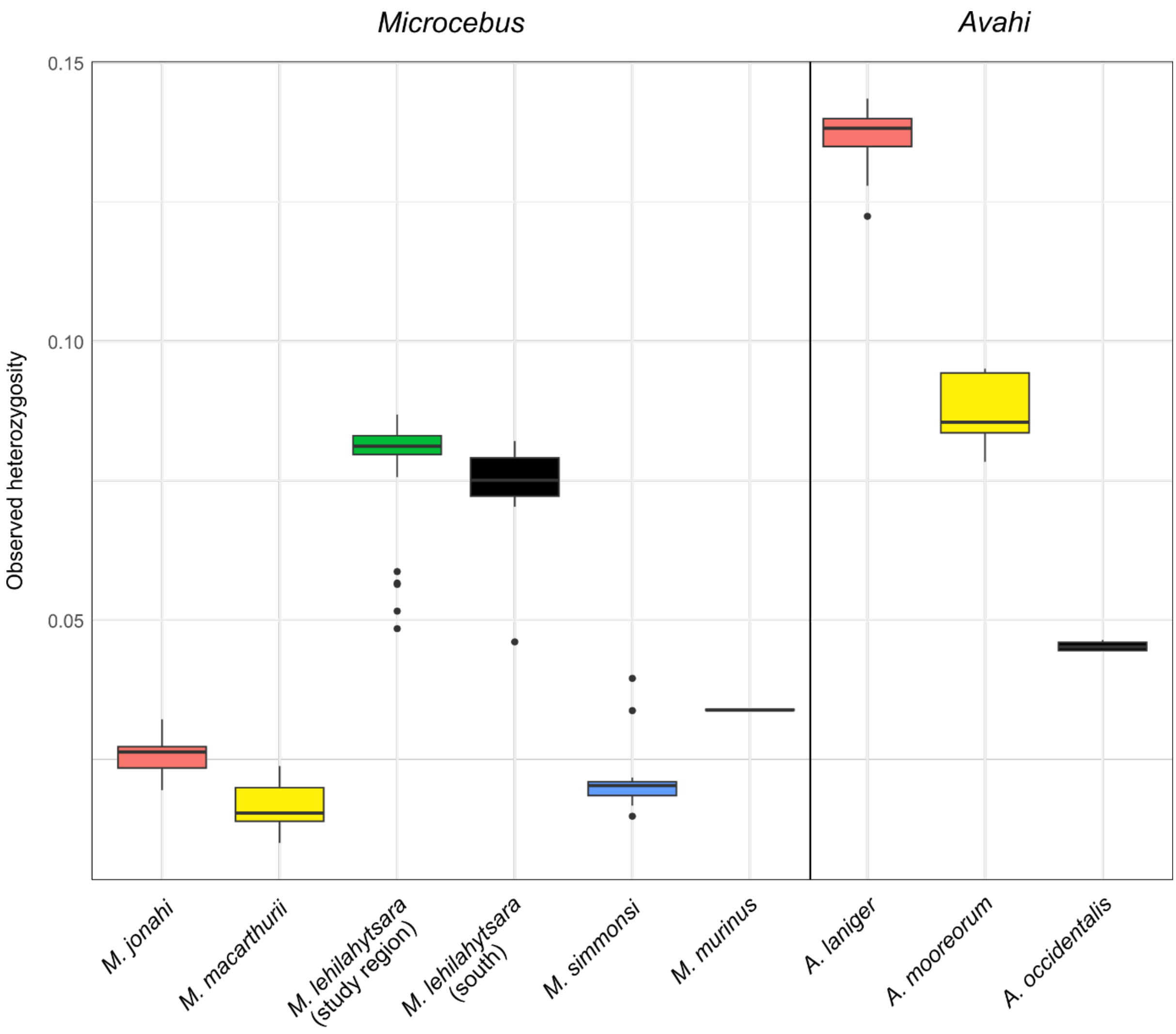


**Fig. S28:** Observed heterozygosity of *Microcebus* spp. and *Avahi* spp. individuals in the study region. Estimates for *M. lehilahytsara* populations south of the region as well as the *M. murinus* and *A. occidentalis* outgroups (black) are added for reference. Significance levels of pairwise comparisons estimated through Dunn's (*post hoc*) test after a Kruskal-Wallis test are given in Table S13. Sample sizes: $n_{M.\ jonahi}$ = 178; $n_{M.\ macarthurii}$ = 15; $n_{M.\ lehilahytsara\ (study\ region)}$ = 82; $n_{M.\ lehilahytsara\ (south)}$ = 31; $n_{M.\ simmonsi}$ = 36; $n_{M.\ murinus}$ = 3; $n_{A.\ laniger}$ = 27; $n_{A.\ mooreroum}$ = 5; $n_{A.\ occidentalis}$ = 4.

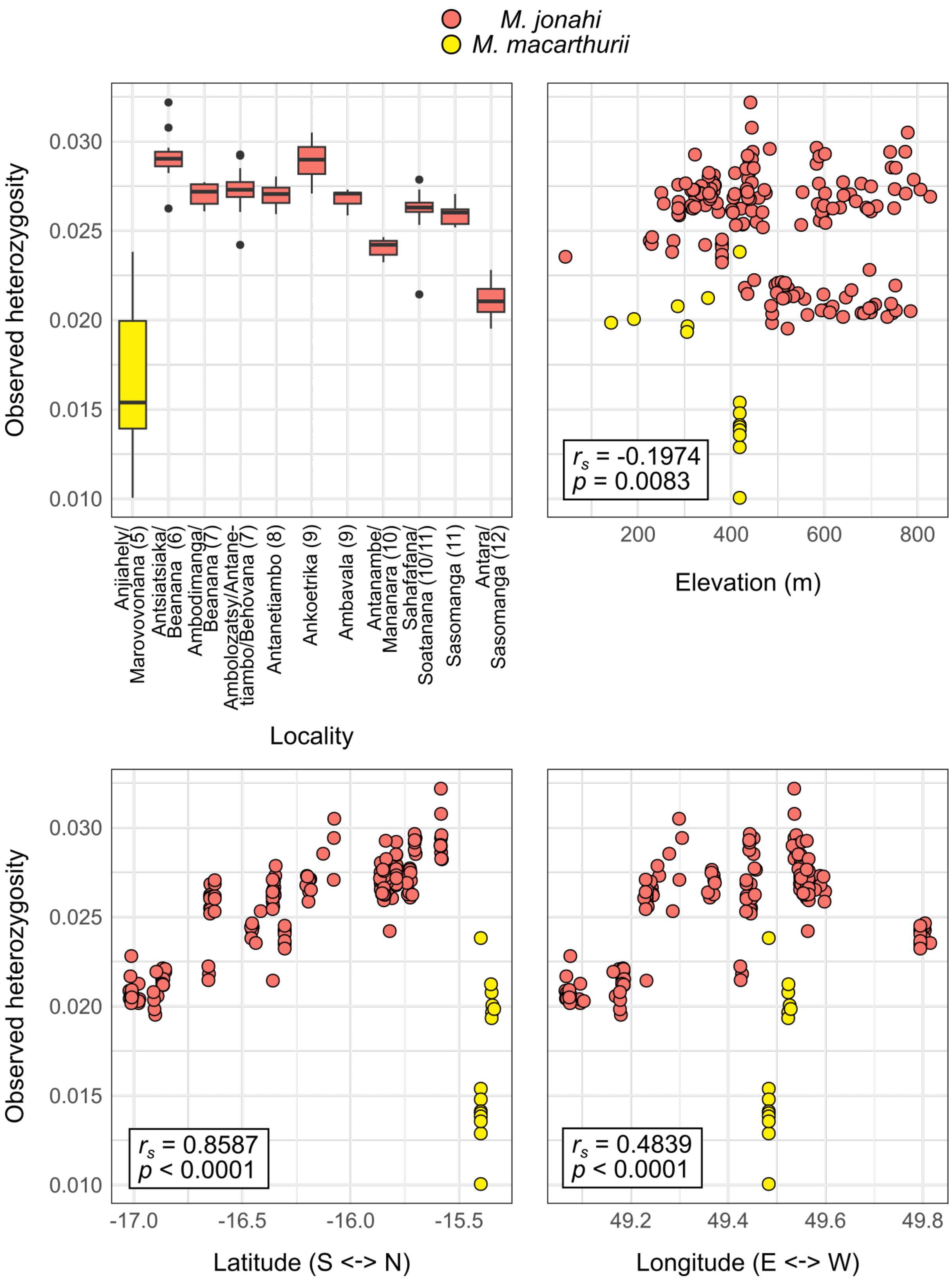


**Fig. S29:** Observed heterozygosity of *M. jonahi* and *M. macarthurii* individuals in the study region in northeastern Madagascar plotted per sampling locality (numbers denote inter-river system) and against elevation, latitude and longitude. Results of Spearman's rank correlation performed on *M. jonahi* data are given in the inlets. S: south; N: north; E: east; W: west. Sample sizes per population can be seen in Table S1.

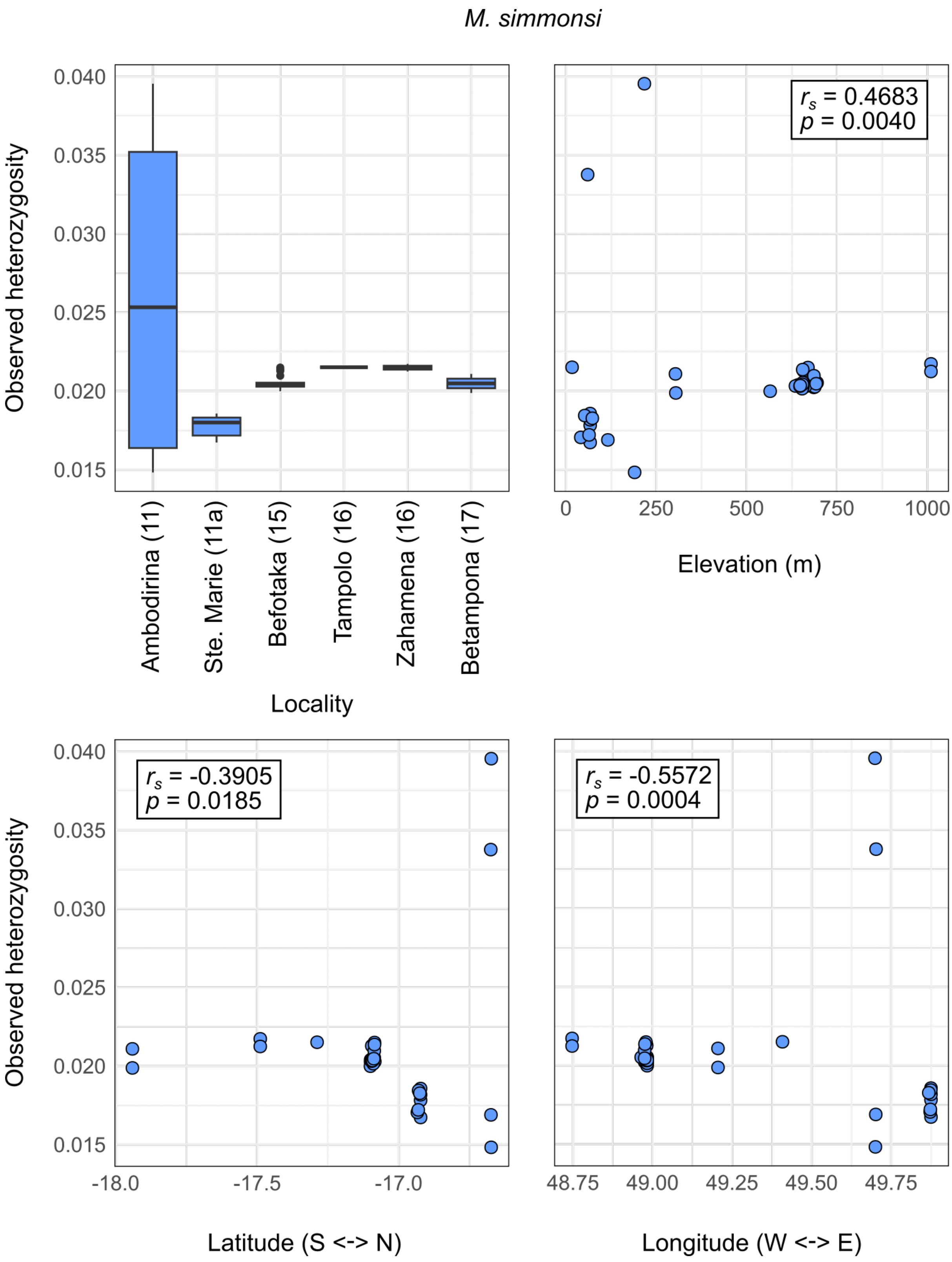


**Fig. S30:** Observed heterozygosity of *M. simmonsi* individuals in the study region in northeastern Madagascar plotted per sampling locality (numbers denote inter-river system) and against elevation, latitude and longitude. Results of Spearman's rank correlation are given in the inlets. S: south; N: north; E: east; W: west. Sample sizes per population can be seen in Table S1.

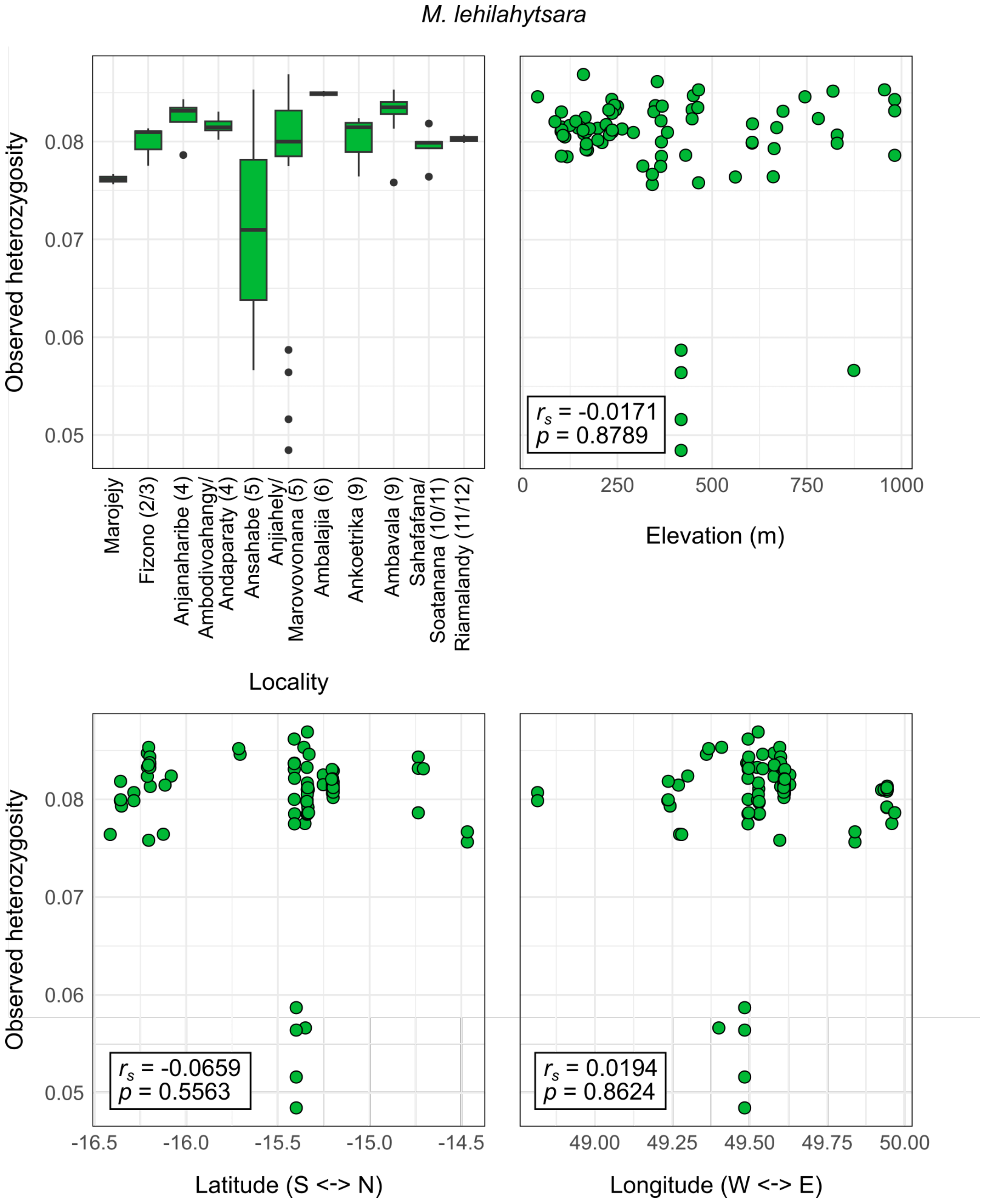


**Fig. S31:** Observed heterozygosity of *M. lehilahytsara* individuals in the study region in northeastern Madagascar plotted per sampling locality (numbers denote inter-river system) and against elevation, latitude and longitude. Results of Spearman's rank correlation are given in the inlets. The five outliers with observed heterozygosities below 0.06 are samples from Anjiahely (IRS5) and have comparably high proportions of missing data (Tables S2, S12). S: south; N: north; E: east; W: west. Sample sizes per population can be seen in Table S1.

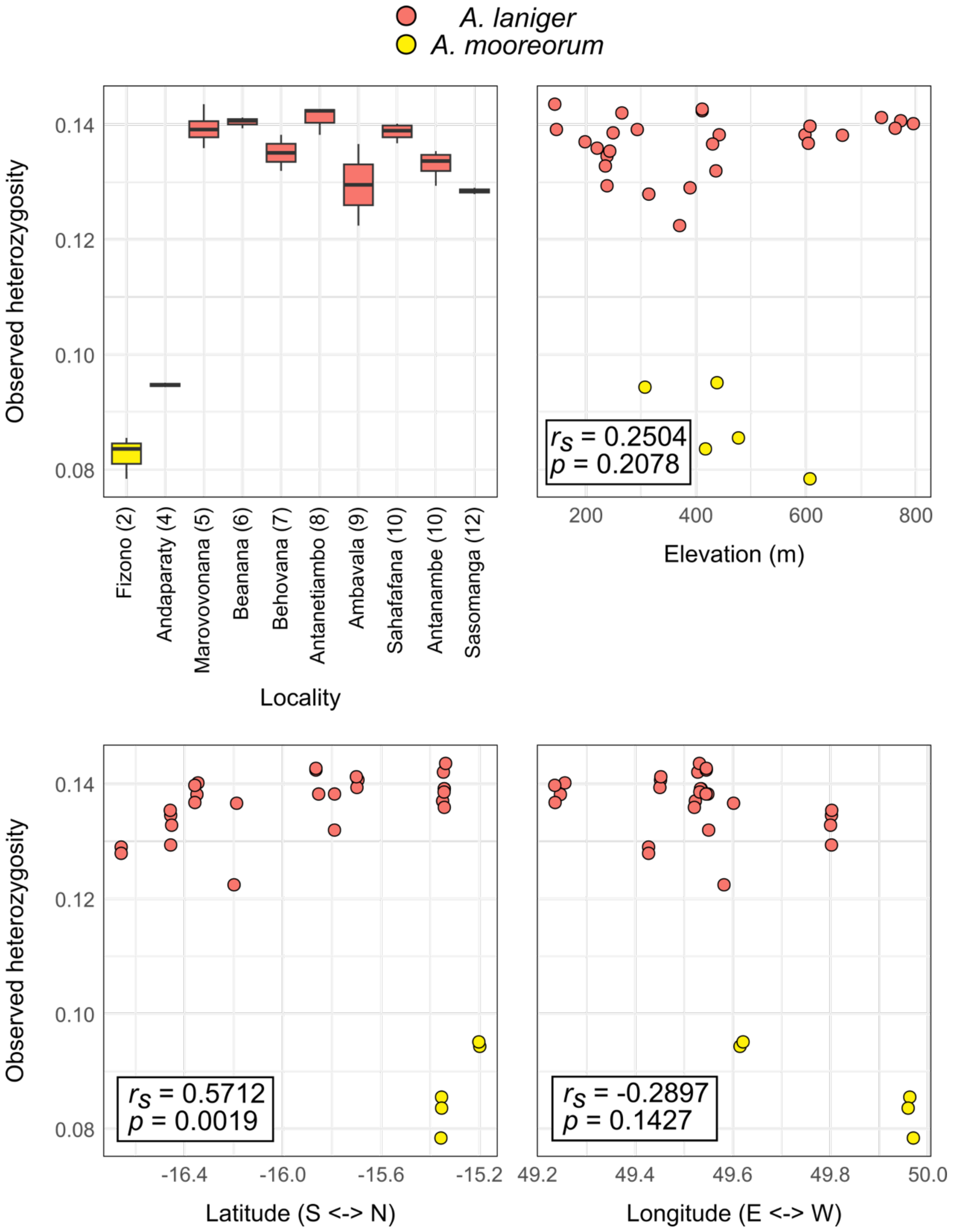


**Fig. S32:** Observed heterozygosity of *A. laniger* and *A. mooreorum* individuals in the study region in northeastern Madagascar plotted per sampling locality (numbers denote inter-river system) and against elevation, latitude and longitude. Results of Spearman's rank correlation performed on *A. laniger* data are given in the inlets. S: south; N: north; E: east; W: west. Sample sizes per population can be seen in Table S1.

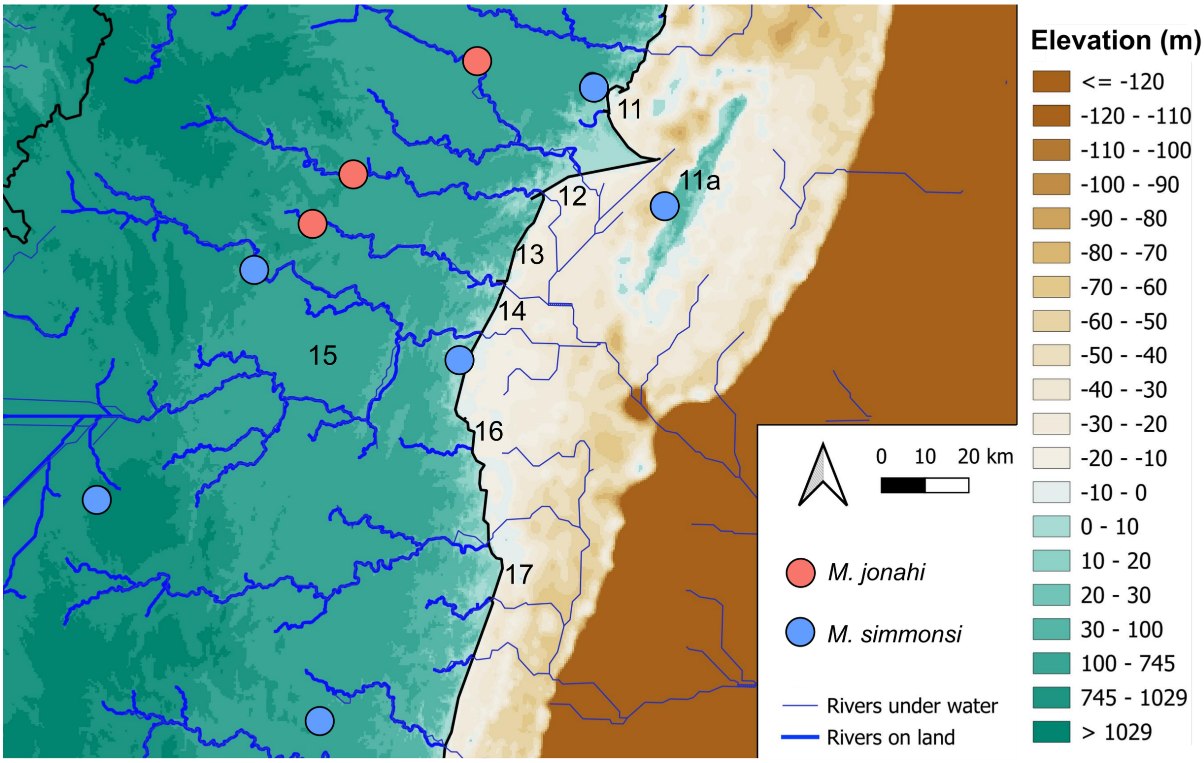


**Fig. S33:** River courses below sea level predicted via flow accumulation from a digital elevation model (Schüßler, 2025). According to the prediction, rivers separating inter-river systems (IRSs) 12–14 (indicated by numbers) converged into a single river when sea levels were lower than today (e.g., during the Last Glacial Maximum), potentially facilitating dispersal of *M. simmonsi* populations from the southern part of its distribution to Île Ste. Marie (IRS 11a). Colored dots indicate sites sampled in this study.

## 4 Supplementary references

Ali, O. A., O'Rourke, S. M., Amish, S. J., Meek, M. H., Luikart, G., Jeffres, C., & Miller, M. R. (2016). Rad capture (Rapture): Flexible and efficient sequence-based genotyping. *Genetics*, *202*(2), 389–400. https://doi.org/10.1534/genetics.115.183665

Andriamasimanana, R. H., Rabenandrasana, M. N., Raminoarisoa, V. T. H. S., Sam, T. S., Virginie, M. C., Ratelolahy, F. J., & Rakotonirainy, E. O. (2001). Effets de la fragmentation de la forêt humide sur les populations d'oiseaux et de lémuriens dans le corridor Mantadia-Zahamena. *Lemur News*, *6*, 18–22.

Danecek, P., Auton, A., Abecasis, G., Albers, C. A., Banks, E., DePristo, M. A., Handsaker, R. E., Lunter, G., Marth, G. T., Sherry, S. T., McVean, G., Durbin, R., & Group, 1000 Genomes Project Analysis. (2011). The variant call format and VCFtools. *Bioinformatics*, 27(15), 2156–2158. https://doi.org/10.1093/BIOINFORMATICS/BTR330

Fonseca, E. M., Pope, N. S., Peterman, W. E., Werneck, F. P., Colli, G. R., & Carstens, B. C. (2024). Genetic structure and landscape effects on gene flow in the Neotropical lizard *Norops brasiliensis* (Squamata: Dactyloidae). *Heredity, 132*, 284–295. https://doi.org/10.1038/s41437-024-00682-5

Genomic Resources Development Consortium, Blanchet, S., Bouchez, O., Chapman, C. A., Etter, P. D., Goldberg, T. L., Johnson, E. A., Jones, J. H., Loot, G., Omeja, P., Rey, O., Ruiz-Lopez, M. J., Switzer, W. M., & Ting, N. (2015). Genomic resources notes accepted 1 December 2014 - 31 January 2015. *Molecular Ecology Resources*, *15*(3), 684–684. https://doi.org/10.1111/1755-0998.12388

Kamilar, J. M., Blanco, M. B. B., & Muldoon, K. M. (2016). Ecological niche modeling of mouse lemurs (*Microcebus* spp.) and its implications for their species diversity and biogeography. In *The Dwarf and Mouse Lemurs of Madagascar: Biology, Behavior and Conservation Biogeography of the Cheirogaleidae* (pp. 449–460). Cambridge University Press.

Karger, D. N., Conrad, O., Böhner, J., Kawohl, T., Kreft, H., Soria-Auza, R. W., Zimmermann, N. E., Linder, H. P., & Kessler, M. (2017). Climatologies at high resolution for the Earth's land surface areas. *Scientific Data*, *4*, 170122. https://doi.org/10.1038/sdata.2017.122

Kass, J. M., Muscarella, R., Galante, P. J., Bohl, C. L., Pinilla-Buitrago, G. E., Boria, R. A., Soley-Guardia, M., & Anderson, R. P. (2021). ENMeval 2.0: Redesigned for customizable and reproducible modeling of species' niches and distributions. *Methods in Ecology and Evolution*, *12*(9), 1602–1608. https://doi.org/10.1111/2041-210X.13628

Lei, R., Engberg, S., Andriantompohavana, R., McGuire, S. M., Mittermeier, R. A., Zaonarivel, J. R., Brenneman, R. A., & Louis Jr, E. E. L. (2008). Nocturnal lemur diversity at Masoala National Park. *Special Publications, Museum of Texas Tech University*, *53*, 1–41.

Martin, S. H., Davey, J. W., & Jiggins, C. D. (2015). Evaluating the use of ABBA-BABA statistics to locate introgressed loci. *Molecular Biology and Evolution, 32*(1), 244–257. https://doi.org/10.1093/molbev/msu269

McKenna, A., Hanna, M., Banks, E., Sivachenko, A., Cibulskis, K., Kernytsky, A., Garimella, K., Altshuler, D., Gabriel, S., Daly, M., & DePristo, M. A. (2010). The genome analysis toolkit: A MapReduce framework for analyzing next-generation DNA sequencing data. *Genome Research*, 20, 1297–1303. https://doi.org/10.1101/gr.107524.110

Mittermeier, R. A., Reuter, K. E., Rylands, A. B., Louis Jr., E. E., Ratsimbazafy, J. H., Rene de Roland, L.-A., Langrand, O., Schwitzer, C., Johnson, S. E., Godfrey, L. R., Blanco, M. B., Borgerson, C., Eppley, T. M., Andriamanana, T., Volampeno, S., Andriantsaralaza, S., Wright, P. C., &

Rajaobelina, S. (2023). *Lemurs of Madagascar* (R. A. Mittermeier, Ed.; 5th ed.). Conservation International.

O'Leary, S. J., Puritz, J. B., Willis, S. C., Hollenbeck, C. M., & Portnoy, D. S. (2018). These aren't the loci you're looking for: Principles of effective SNP filtering for molecular ecologists. *Molecular Ecology*, 27(16), 3193–3206. https://doi.org/10.1111/mec.14792

Poelstra, J. W., Salmona, J., Tiley, G. P., Schüßler, D., Blanco, M. B., Andriambeloson, J. B., Bouchez, O., Campbell, C. R., Etter, P. D., Hohenlohe, P. A., Hunnicutt, K. E., Iribar, A., Johnson, E. A., Kappeler, P. M., Larsen, P. A., Manzi, S., Ralison, J. M., Randrianambinina, B., Rasoloarison, R. M., … Yoder, A. D. (2021). Cryptic patterns of speciation in cryptic primates: Microendemic mouse lemurs and the multispecies coalescent. *Systematic Biology*, *70*(2), 203–218. https://doi.org/10.1093/sysbio/syaa053

Rakotondravony, R., & Rabenandrasana, M. (2011). Inventaire des lémuriens dans la zone de Pointe à Laree, Soanierana-Ivongo, Région Analanjirofo, Madagascar: Implication pour la conservation. *Lemur News*, *16*, 43–48.

Ralimanana, H., Perrigo, A. L., Smith, R. J., Borrell, J. S., Faurby, S., Rajaonah, M. T., Randriamboavonjy, T., Vorontsova, M. S., Cooke, R. S. C., Phelps, L. N., Sayol, F., Andela, N., Andermann, T., Andriamanohera, A. M., Andriambololonera, S., Bachman, S. P., Bacon, C. D., Baker, W. J., Belluardo, F., … Antonelli, A. (2022). Madagascar's extraordinary biodiversity: Threats and opportunities. *Science, 378*(6623), eadf1466. https://doi.org/10.1126/SCIENCE.ADF1466

Raxworthy, C. J. (1986). The lemurs of Zahamena reserve. *Primate Conservation*, *7*, 46–47.

Rochette, N. C., Rivera-Colón, A. G., & Catchen, J. M. (2019). Stacks 2: Analytical methods for paired-end sequencing improve RADseq-based population genomics. *Molecular Ecology*, 28(21), 4737–4754. https://doi.org/10.1111/mec.15253

Schüßler, D. (2025). *Species conservation in the light of evolutionary processes and a changing environment: The mouse lemurs of northeastern Madagascar* [PhD thesis]. University of Hildesheim.

Schüßler, D., Blanco, M. B., Guthrie, N. K., Sgarlata, G. M., Dammhahn, M., Ernest, R., Evasoa, M. R., Hasiniaina, A., Hending, D., Jan, F., le Pors, B., Miller, A., Olivieri, G., Rakotonanahary, A. N., Rakotondranary, S. J., Rakotondravony, R., Ralantoharijaona, T., Ramananjato, V., Randrianambinina, B., … Radespiel, U. (2023). Morphological variability or inter-observer bias? A methodological toolkit to improve data quality of multi-researcher datasets for the analysis of morphological variation. *American Journal of Biological Anthropology, 183*(1), 60–78. https://doi.org/10.1002/AJPA.24836

Schüßler, D., Blanco, M. B., Salmona, J., Poelstra, J., Andriambeloson, J. B., Miller, A., Randrianambinina, B., Rasolofoson, D. W., Mantilla-Contreras, J., Chikhi, L., Louis, E. E., Yoder, A. D., & Radespiel, U. (2020). Ecology and morphology of mouse lemurs (*Microcebus* spp.) in a hotspot of microendemism in northeastern Madagascar, with the description of a new species. *American Journal of Primatology*, *82*(9), e23180. https://doi.org/10.1002/ajp.23180

Schüßler, D., Bremer, J., Sauerwein, M., & Radespiel, U. (2025). Geomorphological river characteristics explain species turnover in amphibians, reptiles and lemurs in Madagascar's eastern rainforests. *Journal of Biogeography, 52*(5), e15109. https://doi.org/10.1111/JBI.15109

Schüßler, D., van Elst, T., Rabemananjara, N. R., Radriarimanga, T., Rafamantanantsoa, S. M., Randimbiharinirina, R. D., Rasolondraibe, E., Mantilla-Contreras, J., & Radespiel, U. (2025). Ecological plasticity explains the distribution of sympatric and allopatric mouse lemurs

(Microcebus spp.) in northeastern Madagascar. *bioRxiv*, 2025.03.24.645043. https://doi.org/10.1101/2025.03.24.645043

Sordyl, T. (2022). *Geoarchive versus Klimamodelle - Variabilität von historischen Klimamodellen in Madagaskar: Ein Vergleich unterschiedlicher Modellierungen mit verfügbaren Geoarchiven* [MSc thesis]. University of Hildesheim.

Tiley, G. P., van Elst, T., Teixeira, H., Schüßler, D., Salmona, J., Blanco, M. B., Ralison, J. M., Randrianambinina, B., Rasoloarison, R. M., Stahlke, A. R., Hohenlohe, P. A., Chikhi, L., Louis, E. E., Radespiel, U., & Yoder, A. D. (2022). Population genomic structure in Goodman's mouse lemur reveals long-standing separation of Madagascar's Central Highlands and eastern rainforests. *Molecular Ecology*, *31*(19), 4901–4918. https://doi.org/10.1111/mec.16632

Tournebize, R., & Chikhi, L. (2025). Ignoring population structure in hominin evolutionary models can lead to the inference of spurious admixture events. *Nature Ecology and Evolution, 9*, 225–236. https://doi.org/10.1038/s41559-024-02591-6

Tricou, T., Tannier, E., & de Vienne, D. M. (2022). Ghost lineages highly influence the interpretation of introgression tests. *Systematic Biology, 71*(5), 1147–1158. https://doi.org/10.1093/sysbio/syac011

van Elst, T., Sgarlata, G. M., Schüßler, D., Tiley, G. P., Poelstra, J. W., Scheumann, M., Blanco, M. B., Aleixo-Pais, I. G., Rina Evasoa, M., Ganzhorn, J. U., Goodman, S. M., Hasiniaina, A. F., Hending, D., Hohenlohe, P. A., Ibouroi, M. T., Iribar, A., Jan, F., Kappeler, P. M., Le Pors, B., … Salmona, J. (2025). Integrative taxonomy clarifies the evolution of a cryptic primate clade. *Nature Ecology & Evolution*, *9*, 57–72. https://doi.org/10.1038/s41559-024-02547-w

Vieilledent, G., Grinand, C., Rakotomalala, F. A., Ranaivosoa, R., Rakotoarijaona, J. R., Allnutt, T. F., & Achard, F. (2018). Combining global tree cover loss data with historical national forest cover maps to look at six decades of deforestation and forest fragmentation in Madagascar. *Biological Conservation*, *222*, 189–197. https://doi.org/10.1016/j.biocon.2018.04.008

Warren, D. L., Matzke, N. J., Cardillo, M., Baumgartner, J. B., Beaumont, L. J., Turelli, M., Glor, R. E., Huron, N. A., Simões, M., Iglesias, T. L., Piquet, J. C., & Dinnage, R. (2021). ENMTools 1.0: An R package for comparative ecological biogeography. *Ecography*, *44*(4), 504–511. https://doi.org/10.1111/ECOG.05485

Wilmé, L., Goodman, S. M., & Ganzhorn, J. U. (2006). Biogeographic evolution of Madagascar's microendemic biota. *Science, 312*(5776), 1063–1065. https://doi.org/10.1126/science.1122806
